# Structural effects of arsine ligands on C–H difunctionalization of thiophene

**DOI:** 10.1039/d5sc05285h

**Published:** 2025-10-10

**Authors:** Akifumi Sumida, Kaisei Yamamoto, Takahiro Iwamoto, Kensuke Naka, Hiroaki Imoto

**Affiliations:** a Faculty of Molecular Chemistry and Engineering, Kyoto Institute of Technology Goshokaido-cho, Matsugasaki, Sakyo-ku Kyoto 606-0962 Japan himoto@kit.ac.jp; b Materials Innovation Lab, Kyoto Institute of Technology Goshokaido-cho, Matsugasaki, Sakyo-ku Kyoto 606-0962 Japan; c Fusion Oriented REsearch for Disruptive Science and Technology (FOREST), Japan Science and Technology Corporation (JST) Honcho 4-1-8 Kawaguchi Saitama 332-0012 Japan

## Abstract

Despite their significant potential in organometallic chemistry, the utility of arsines as ligands in transition-metal catalysis remains underexplored relative to their phosphine counterparts. Although the Pd-catalyzed C–H difunctionalization of thiophene proceeds efficiently with triphenylarsine (AsPh_3_) but fails with conventional phosphine ligands, the synthetic utility of arsine ligands other than AsPh_3_ has not been explored. In this study, the steric and electronic requirements of the Pd-catalyzed C–H difunctionalization of thiophene are explored using 36 synthesized arsines and nine phosphines. Ligand parameterization reveals that arsines with moderate electron-donating abilities and sufficient steric accessibility were preferred. Notably, the identified steric demand is more readily met by arsines than by phosphines. Furthermore, arsines exhibit superior oxidative stability under reaction conditions that typically oxidize phosphines owing to the high oxophilicity of phosphorus. These experimental and computational findings demonstrate that the use of arsines can expand the scope of transition metal catalysts by enabling access to catalytic spaces that are less accessible with traditional phosphines.

## Abstract 2

This study highlights the advantages of arsine ligands over phosphines in Pd-catalyzed C–H difunctionalization. Analysis of 36 arsines shows higher steric accessibility and oxidative stability, enabling new catalyst designs beyond phosphine limits.

## Graphical abstract

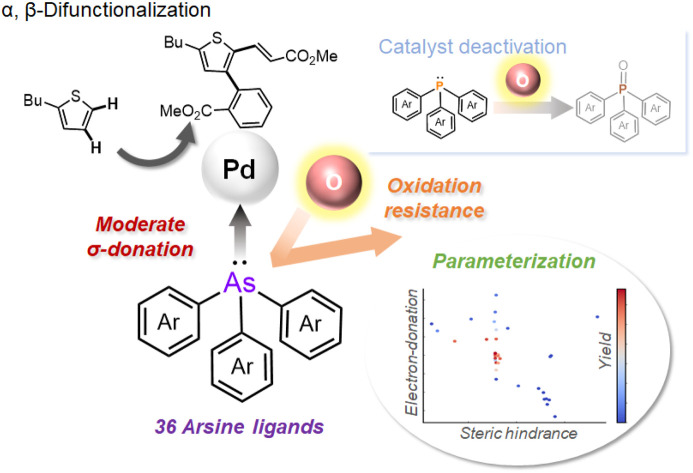

## Introduction

Since the discovery of trimethylphosphine in 1847 by Paul Thénard,^1^ various phosphine derivatives have been developed, significantly advancing the field of coordination chemistry.^2^ In particular, phosphine ligands are crucial in homogeneous transition metal-catalyzed reactions such as coupling reactions^3^ and asymmetric hydrogenation.^4^ The chemical structures of phosphine ligands optimize their catalytic activity and selectivity. The steric and electronic parameters of various phosphine ligands have been examined to understand the structure–activity relationship of the catalyst.^5^ For instance, Tolman introduced the cone angle^5*a*,*c*^ and Tolman electronic parameter (TEP)^5*b*,*c*^ to estimate the steric bulk and electron-donating abilities of tertiary phosphine ligands, respectively. Additionally, the percent buried volume (%*V*_bur_) was introduced to evaluate steric congestion around the metal center.^5*d*,*e*^ These parameters have been instrumental in facilitating the design of suitable ligand structures.

Arsine ligands are promising alternatives to phosphorus ligands in transition-metal catalysis.^6^ Arsenic-based ligands typically have higher TEP than their phosphorus-based counterparts owing to the weaker σ-donating ability of arsenic.^7^ Moreover, metal–arsenic bonds are typically longer than metal-phosphorus bonds owing to the larger atomic radius of arsenic, resulting in a more sterically open environment around the metal center that results in smaller cone angles and reduced %*V*_bur_ values.^8^ In addition, the high oxidative resistance of arsine ligands allows the catalyst to retain its activity in the presence of oxygen, thus enabling coupling reactions under air rather than requiring a nitrogen atmosphere.

**Fig. 1:**
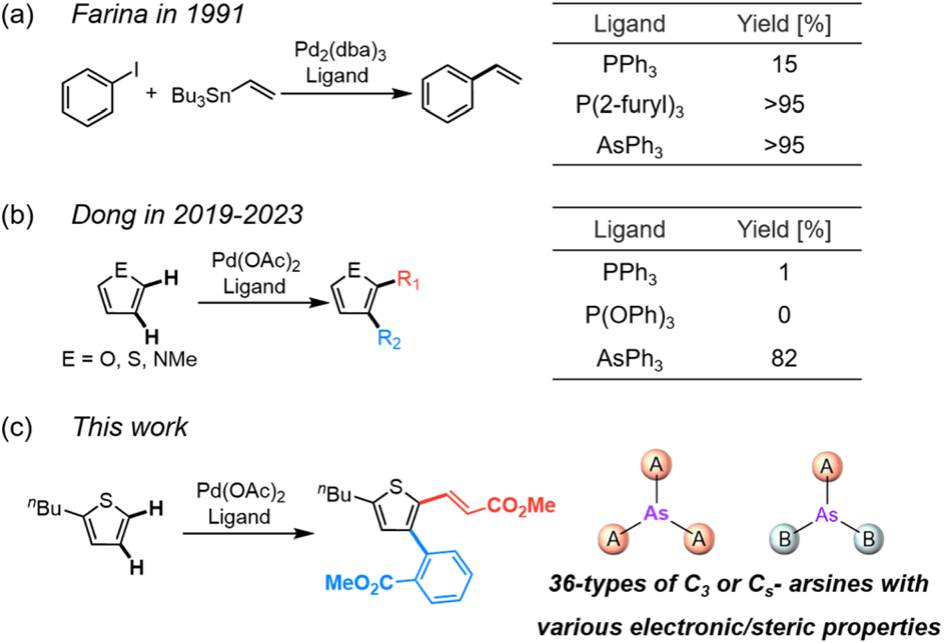
Reactions promoted by arsine-ligated transition metal catalysts: (a) Stille coupling and (b and c) C–H functionalization of thiophene using triphenylarsine and 36 arsine ligands (c, this work).

In 1991, Farina demonstrated that substituting triphenylphosphine (PPh_3_, L28) with triphenylarsine (AsPh_3_, L1) significantly catalyzed the Stille coupling reaction (Fig. 1a).^6*a*^ This behavior is similar to that of tri(2-furyl)phosphine, a weakly σ-donating phosphine. Weak σ-donation promotes the rate-determining dissociation of the ligand from the Pd(ii) center, thereby accelerating the reaction. Despite these promising characteristics, research on arsine ligands beyond L1 is limited.^9^ Understanding the structure–activity relationship of arsine ligands is expected to greatly advance the field of transition metal catalysis. This limited exploration is attributed to the challenges associated with conventional synthetic routes, which typically require volatile and toxic arsenic precursors such as arsenic chlorides and hydrides.^10^ Synthetic methods using nonvolatile arsenic precursors have been developed to address this limitation.^11^ For example, various organoarsenic compounds were synthesized *via* the conversion of cyclooligoarsines (cyclopentamethylpentaarsine and cyclohexaphenylhexaarsine) and arsenic trioxide (As_2_O_3_) into arsenic radicals,^11*a*^ electrophiles,^11*b*,*d-f*^ and nucleophiles,^11*c*^ establishing a comprehensive library of arsenic ligands to facilitate the study of their structure–activity relationships in Pd-catalyzed coupling reactions. Arsenic ligands have advantages over traditional phosphine ligands in catalytic reactions.^8,11*f*,12^

Recently, Dong *et al.* developed cooperative palladium/norbornene (Pd/NBE) catalysts for C–H functionalization (Fig. 1b).^13^ Interestingly, some of these reactions are only feasible with L1, because even phosphine ligands with weakly σ-donating substituents show no activity. This behavior contrasts with the aforementioned Stille coupling reaction and suggests the existence of reactions that can only be realized using arsenic ligands; however, the specific structural features of the arsine ligands that enable these reactions are poorly understood because previous studies were limited to the commercially available arsine ligand L1. Moreover, although the difunctionalization of indoles has been studied using computational methods,^13*e*^ the failure of the difunctionalization reaction in the presence of the phosphine-ligated catalyst remains unexplained, primarily owing to the absence of experimental and computational data. Dong *et al.* explored the mechanism of difunctionalization reactions under various conditions;^13^ however, the role of arsine ligands and the limitations of phosphines were not thoroughly explored. To address these knowledge gaps, we investigated Pd/NBE-catalyzed difunctionalization using diverse arsine and phosphine ligands to elucidate the role of arsenic in this transformation. Understanding these ligand-dependent properties is expected to significantly expand the chemical space accessible *via* transition-metal catalysis.

In this study, 36 arsenic ligands were synthesized using practical methods, and their steric and electronic properties were determined using computational calculations (Fig. 1c). The structure–activity relationship of the catalysts in the Pd-catalyzed C–H functionalization of thiophene was elucidated. Among the nine phosphorus ligands examined, none showed any catalytic activity, while arsine ligands with moderate electron-donating properties and low %*V*_bur_ values exhibited catalytic activity. Density functional theory (DFT) calculations and NMR experiments further elucidated the differences in catalytic activity of the arsine- and phosphine-ligated catalysts.

## Results and discussion

Arsine ligands L2–L27 were synthesized using arsenic tribromide or 1-bromobenzodithiaarsole in accordance with an established procedure.^11*e*,*f*^ For comparison, phosphine ligands L28–L36 were obtained from commercial sources, except for L33, which was synthesized in our laboratory. To parameterize their electronic and steric properties, the structures of L-Ni(CO)_3_ (L = ligand) were optimized using DFT calculations at the B3LYP-D3 level (LANL2DZ for Ni and def2-TZVP for the others).^14^ The electronic parameters were extracted from the optimized structures using frequency calculations and converted to TEP according to a reported equation.^15^ In addition, steric parameters, including the cone angle and %*V*_bur_, were calculated using the Morfeus^16^ and SambVca 2.1 programs (Table S1).^17^

**Scheme 1:**
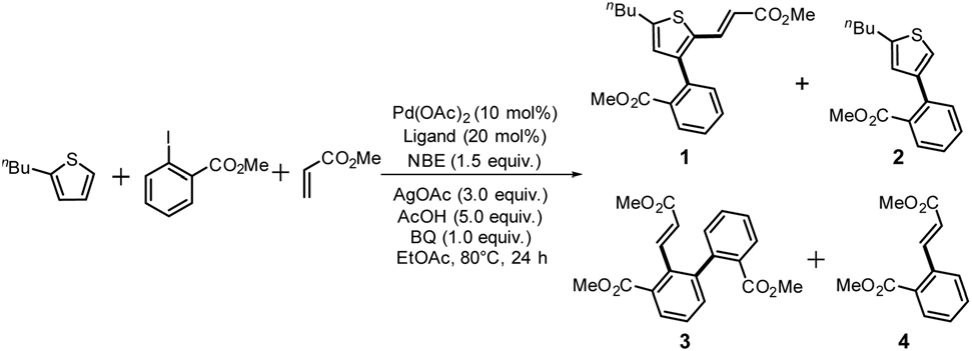
Catalytic C–H difunctionalization reaction of thiophene, yielding the difunctionalized product 1, monofunctionalized product 2, Catellani product 3, and Heck product 4.

**Fig. 2:**
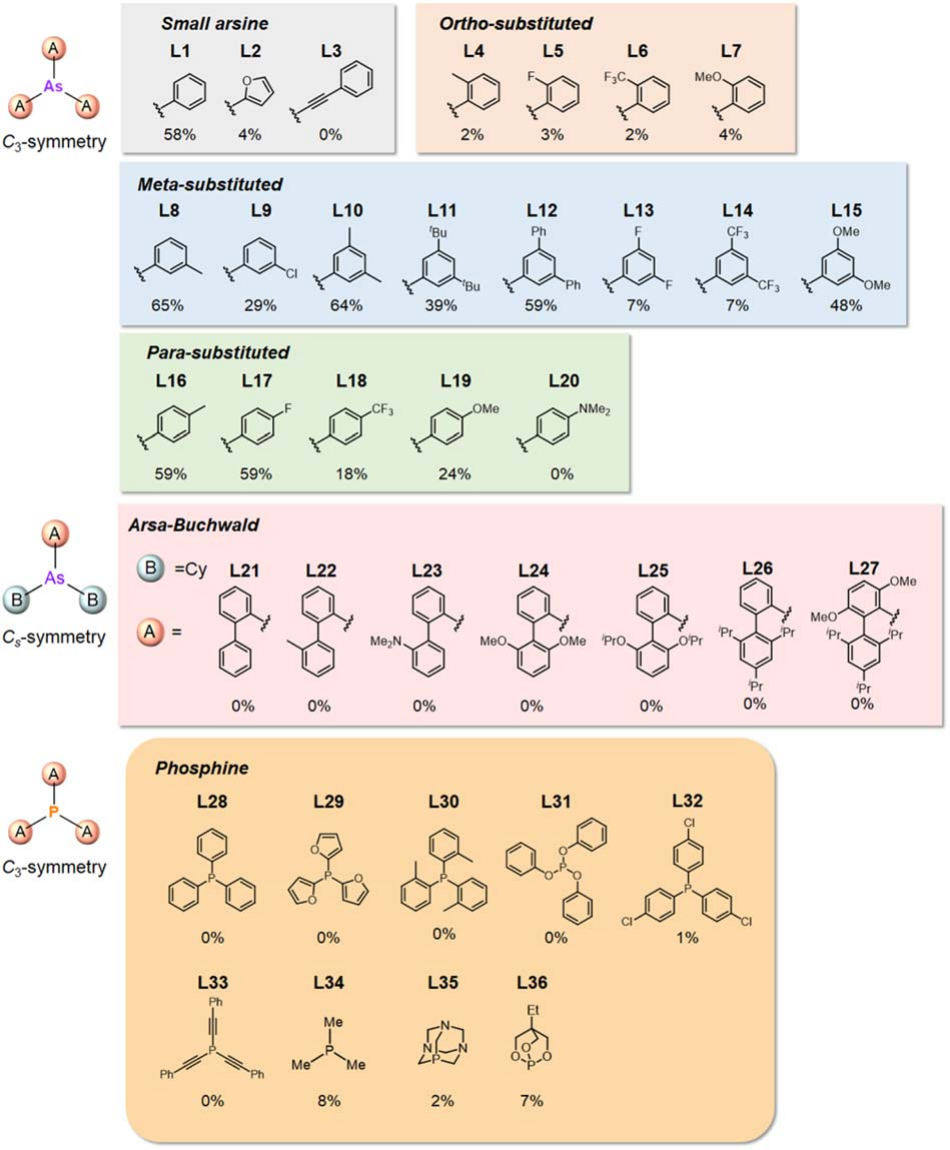
*o*-, *m*-, and *p*-substituted *C*_3_- and *C*_s_-type arsine and phosphine ligands. The NMR yield of the difunctionalized product 1 (Scheme 1) is shown below each structure.

The difunctionalization of thiophene was performed using the Pd/NBE cooperative catalyst with the prepared ligands L1–L36 (Scheme 1).^13*c*^ The reaction conditions were based on a previously reported procedure with slight modifications: an ethyl acetate (EtOAc) solution with palladium acetate (Pd(OAc)_2_, 10 mol%) and a ligand (20 mol%), *N*-methylamide-norbornene (NBE, 1.5 eq.) in the presence of silver acetate (AgOAc, 3.0 eq.), acetic acid (AcOH, 5.0 eq.), and benzoquinone (BQ, 1.0 eq.).^18^ The reaction time and temperature were 24 h and 80 °C, respectively. Four components were identified in the ^1^H-NMR spectra of the crude mixture: α,β-difunctionlized product (1, target compound), β-monofunctionalized product (2), Catellani product (3), and Heck product (4) (Fig. 2). The NMR yields of the desired difunctionalized products 1 obtained using each ligand are summarized in Fig. 1. All yields, including the compositions of 1–4, are listed in Table S1.

A range of triarylarsines (L1–L20) demonstrated moderate to good activity (L1: 58%, L8: 65%, L10: 64%, L12: 59%, L16: 59%, and L17: 59%). In contrast, arsa-Buchwald ligands L21–L27 showed no activity, nor did their electron-rich or sterically hindered derivatives (0%). Similarly, most phosphine ligands (L28–L33) afforded no reaction. Trialkylphosphines L34–L36 yielded minimal amounts of the target products (<8%); thus, the phosphine ligands exhibited negligible catalytic activity in this reaction regardless of their electronic or steric properties, suggesting the involvement of an alternative factor.

**Fig. 3:**
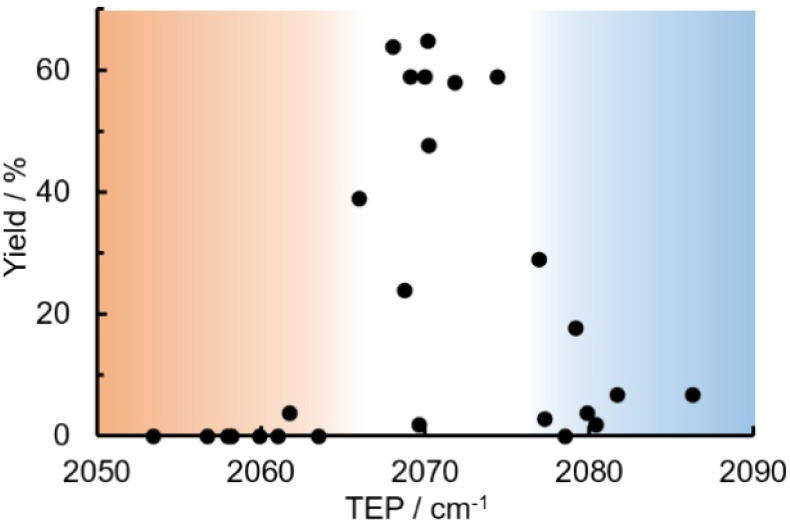
Scatter plot of yield *vs.* TEP. The orange and blue regions indicate the TEP ranges of electron-rich and deficient ligands, respectively.

A scatter plot of the obtained yields against TEP (Fig. 3) revealed the influence of the electronic properties on the reaction, showing that high catalytic activity (>40%) requires arsine ligands with TEP values between 2067–2075 cm^−1^: however, some compounds with TEP values in this range exhibit low activity owing to the steric requirement. To understand the electronic effects, *para*-substituted triarylarsines L1 and L16–L20, which had similar cone angles (158.1–159.6°) and %*V*_bur_s (22.7–22.9%), were evaluated (Table 1). Strongly electron-donating or withdrawing groups, including—trifluoromethyl (L18: 18%, TEP = 2079.1 cm^−1^), methoxy (L19: 24%, TEP = 2068.7 cm^−1^), and dimethylamino (L20: 0%, TEP = 2063.5 cm^−1^) groups, resulted in low reactivity, affording significantly lower yields than that obtained using AsPh_3_ (L1, 58%, TEP = 2071.3 cm^−1^). In contrast, the performance of ligands bearing substituents with moderate electronic effects, including methyl (L16: 59%, TEP = 2069.9 cm^−1^) and fluoro (L17: 59%, TEP = 2074.4 cm^−1^) groups, was similar to that of AsPh_3_ (L1). The highly active ligands L1 and L16 afforded low yields of byproduct 2 (12% and 15%, respectively), indicating that C–H functionalization can occur. The Heck reaction appeared to compete with the protodemetalation step. Under ligand-free conditions, the Heck product 3 (6%) and Catellani product 4 (11%) were obtained.^19^ Moreover, in a thiophene-free control experiment using AsPh_3_ (L1) or PPh_3_ (L28), small amounts of 3 and 4 were also formed, implying that these products are unlikely to originate from intermediates proposed in our catalytic cycle.

**Table 1:** Product of the model reaction with *p*-substituted arsine ligands (AsR_3_), along with the calculated TEPs

R	1/%	2/%	3/%	4/%	TEP/cm^−1^
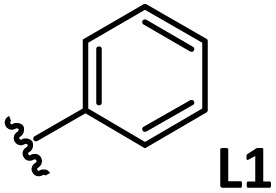	58	12	0	0	2071.3
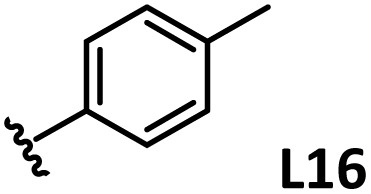	59	15	0	0	2069.9
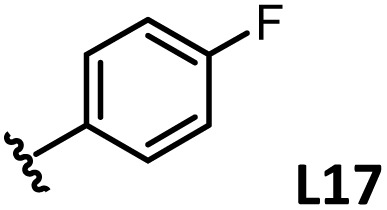	59	7	4	4	2074.4
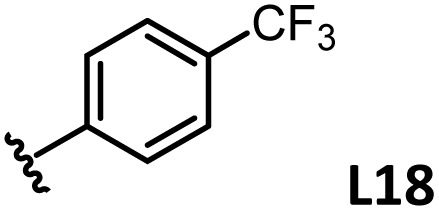	18	4	1	0	2079.1
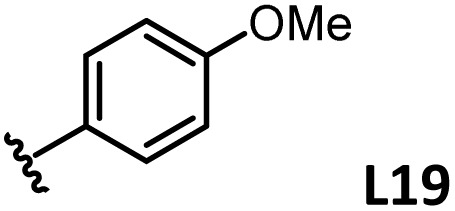	24	5	0	0	2068.7
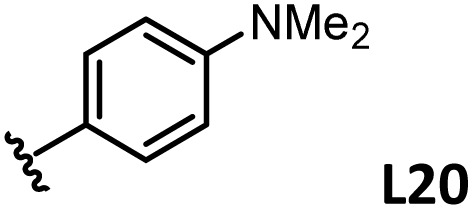	0	0	2	2	2063.5
None	0	0	6	11	—

This tendency was also observed using L20 and PPh_3_ (L28, see Table S1), indicating that such ligands may be deactivated. Time-dependent monitoring of products 1–4 when using L1 (Fig. S3) confirmed that these compounds were generated in parallel, consistent with Dong's reported mechanism.^13*c*^

The interaction of BQ with AsPh_3_ (L1), electron-rich L19, and L20 was examined. The addition of BQ to L20 resulted in an color change from colorless to yellow, whereas the color change upon addition of BQ to a solution of L19 occurred gradually.^20^^1^H-NMR spectra acquired 24 h after the addition of BQ confirmed the formation of charge transfer (CT)-complexes with L19 and L20. In contrast, AsPh_3_ (L1) retained its original appearance and NMR profile. These observations suggest that BQ and electron-rich ligands form CT complexes that inhibit C–H activation, thereby reducing the performance of the catalyst. Furthermore, the concerted metalation–deprotonation (CMD) step favors electron-deficient ligands because the highly electrophilic Pd center strongly interacts with the heteroarene moiety. Indeed, electron-deficient arsine ligands tend to facilitate the CMD step in the direct arylation of thiophenes. However, strongly electron-deficient thioether ligands are known to be ineffective for the oxidative coupling of thiophenes, as excessive electron deficiency may suppress the initial C–H palladation.^21^ In addition, electron-deficient arsine ligands can coordinate only weakly, resulting in a labile Pd–ligand bond and potential discoordination.^12*a*^ Therefore, moderately electron-deficient arsine ligands appear optimal for the present reaction.

The performances of AsPh_3_ (L1), *o*-tolyl (L4), *m*-tolyl (L8), and *p*-tolyl (L16) arsines were compared to determine the steric effects of the ligands (Table 2). The TEP values of these ligands fall within the favorable range (2069.6–2070.1 cm^−1^). Ligands with *o*-substituents result in steric hindrance around the metal, with large cone angles and %*V*_bur_ values, whereas *m*-substituents have remote steric hindrance, with large cone angles and moderate %*V*_bur_ values. The *o*-substituted arsine L4 exhibited significantly lower activity (2%) than AsPh_3_ (L1, 58%), L8 (65%), and L16 (59%). The yield obtained using L8 was sufficient compared to those of AsPh_3_ (L1) and L16. These observations imply that the catalytic activity is dependent on %*V*_bur_, although the cone angle does not determine the outcome. Accordingly, the steric hindrance around the Pd center should be controlled under the present reaction conditions, regardless of remote steric hindrance. Sterically hindered indoles or thiophenes typically show lower reactivity,^13*e*^ indicating a high dependency on the steric environment around the metal center. This hypothesis was supported by the high yields obtained using *m*-substituted arsines L10 (64%) and L12 (59%), which have low %*V*_bur_ values (22.9% and 23.3%, respectively) despite their large cone angles (170.5° and 197.8°, respectively). The strongly electron-donating or electron-withdrawing groups of L11, L13, L14, and L15 caused mismatch of the TEP values (L11: 2066.0 cm^−1^, L13: 2081.7 cm^−1^, L14: 2086.3 cm^−1^, and L15: 2070.2 cm^−1^), resulting in relatively low yields (7–48%).

**Table 2:** Products of the model reaction with arsine ligands (AsR_3_), and calculated cone angles and %*V*_bur_s

R	1/%	2/%	3/%	4/%	Cone angle/°	%*V*_bur_/%
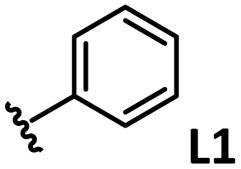	58	12	0	0	158.7	22.8
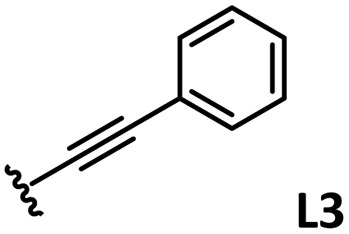	0	0	5	5	162.9	16.2
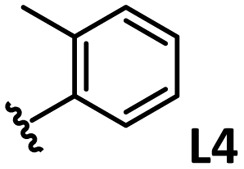	2	4	2	7	180.3	28.5
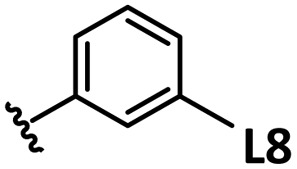	65	12	3	0	161.8	22.8
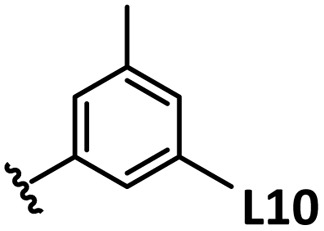	64	13	2	4	170.5	22.9
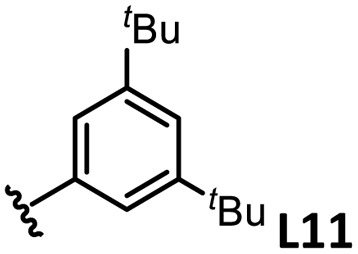	39	11	6	11	195.5	22.9
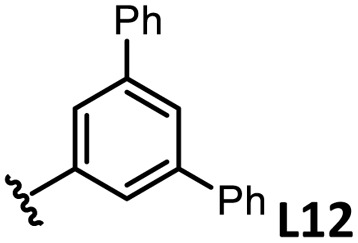	59	11	4	0	197.8	23.3

**Fig. 4:**
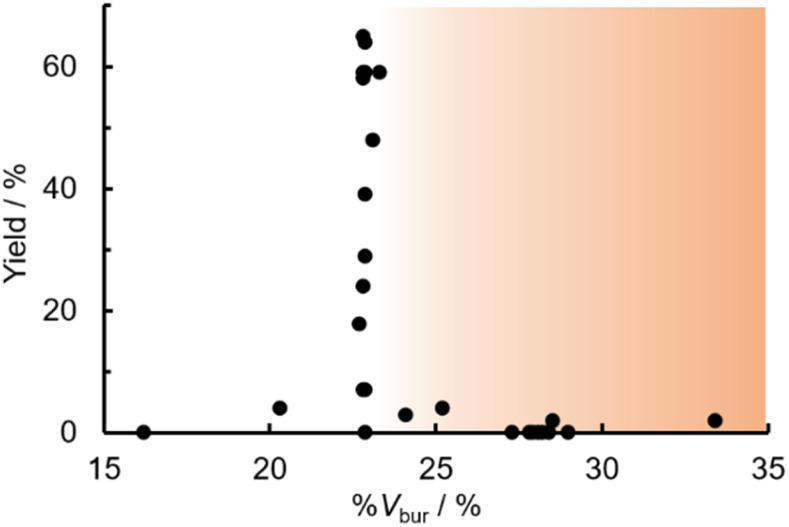
Scatter plot of yield *vs.* %*V*_bur_. The orange region indicates the %*V*_bur_ range of sterically hindered ligands.

The relationship between yield and %*V*_bur_ was analyzed to further elucidate the steric influence of the ligands (Fig. 4). Notably, a critical %*V*_bur_ threshold emerged at approximately 23%, beyond which ligands exhibited minimal activity (<8%). Ligands with very low %*V*_bur_ values, such as ethynylbenzene-substituted L3 (%*V*_bur_ = 16.2%), resulted in negligible catalytic activity (0%). In addition to the mismatched TEP value (2078.5 cm^−1^) of L3, ligands with minimal steric hindrance tended to form stable complexes with a higher number of coordinated ligands (*e.g.*, PdL_4_), which inhibited ligand dissociation.^5*c*,22^ Consequently, ligands with very low %*V*_bur_ values may resist ligand exchange with sterically hindered substrates such as norbornene. We therefore concluded that the C–H difunctionalization of thiophene requires ligands with moderately weak electron-donating properties (TEP = 2067–2075 cm^−1^) and limited steric bulk around the Pd center (%*V*_bur_ ≤ 23%): however, the phosphine ligand L33, which possesses nearly optimal properties (TEP = 2068.7 cm^−1^, %*V*_bur_ = 23.7%), exhibited no catalytic activity (0%), indicating that additional factors influence the catalytic performance of phosphine ligands.

**Fig. 5:**
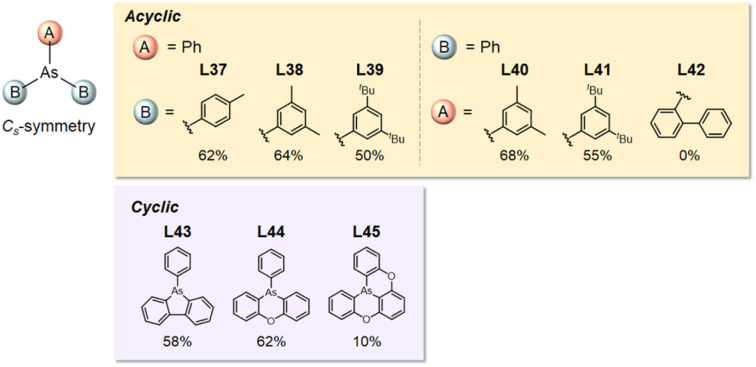
*o*-, *m*-, and *p*-substituted *C*_s_-type arsine ligands. NMR yields are shown below the structures.

These findings prompted us to develop additional arsine ligands with two distinct substituents at the arsenic center (AB_2_-type arsines), enabling the electronic and steric properties of the catalyst to be precisely tuned using various substituent combinations. AB_2_-type arsines containing mono- or diphenyl substituents (L37–L45; Fig. 5) were synthesized and evaluated. The substitution of one L16, L10, and L11 ligand with a phenyl group yielded L37, L38, and L39, respectively. Two substituents of L10 and L11 were substituted with phenyl groups to obtain L40 and L41. L40 (68%) showed the highest activity. No activity was observed in the presence of an *o*-substituted ligand (L42). To reduce steric hindrance, the phenyl groups in L43–L45 were bridged. Interestingly, the ligands with two bridged phenyl groups showed high activity (L43: 58%, L44: 62%), whereas those with three bridged phenyl groups showed significantly lower activity (L45: 10%). Considering their %*V*_bur_ (L43: 18.6%, L44: 21.9%, L45: 16.8%), the threshold to promote the reaction is lower at approximately 18.6%.

**Fig. 6:**
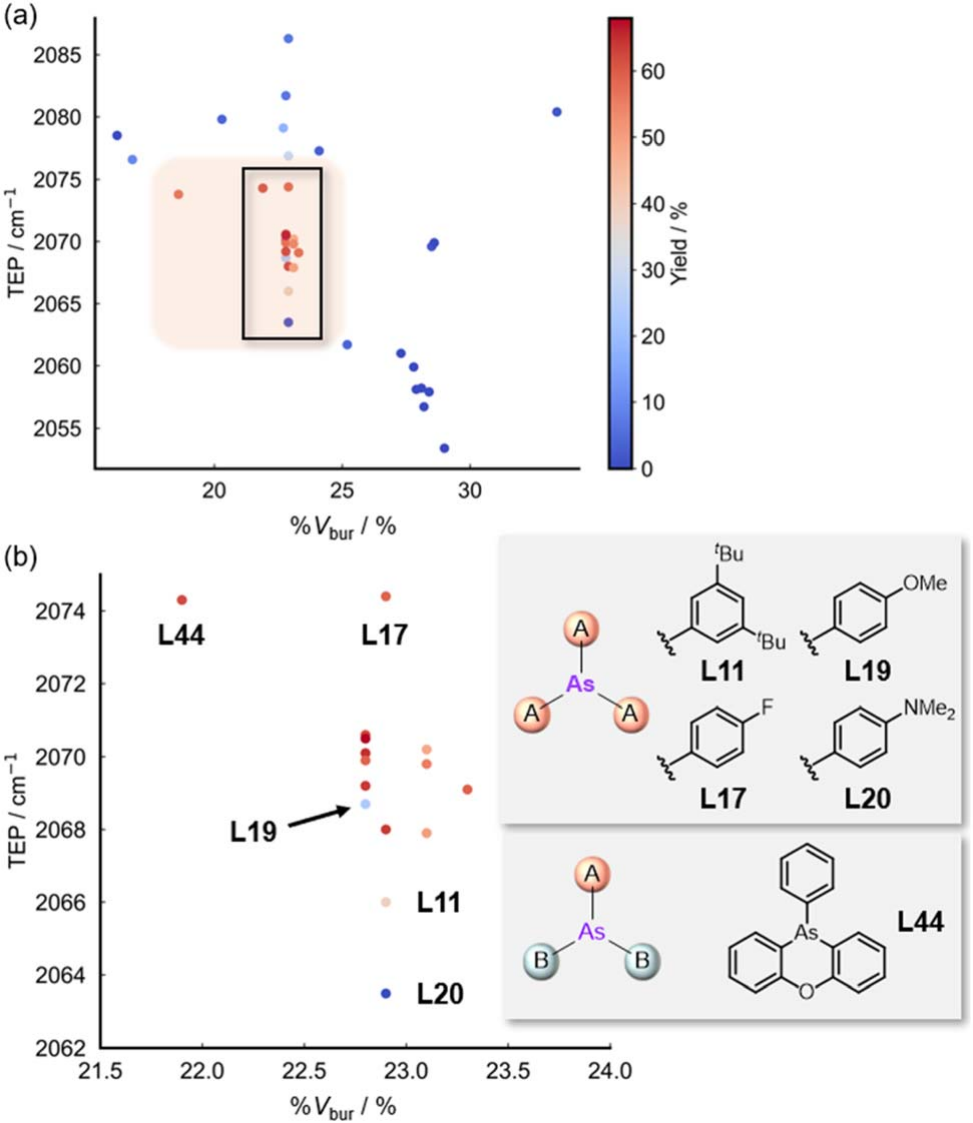
(a) Heatmap of the relationship of TEP *vs.* %*V*_bur_ and yield, and (b) magnified image of the area enclosed by the black rectangle in the high yield region.

A heatmap illustrating the relationship between the TEP values, %*V*_bur_, and catalytic yields was constructed based on our accumulated findings (Fig. 6). Ligands possessing TEP values between 2066–2075 cm^−1^ and %*V*_bur_ values between 21.5–23.5% exhibited optimal catalytic performance. L44 defines the lower boundary for %*V*_bur_ while approaching the upper limit of the TEP values. Notably, despite falling within this high-activity region, L19 afforded a modest yield (24%) owing to ligand deactivation (for detail of the ligand deactivation, see Fig. S2).

**Fig. 7:**
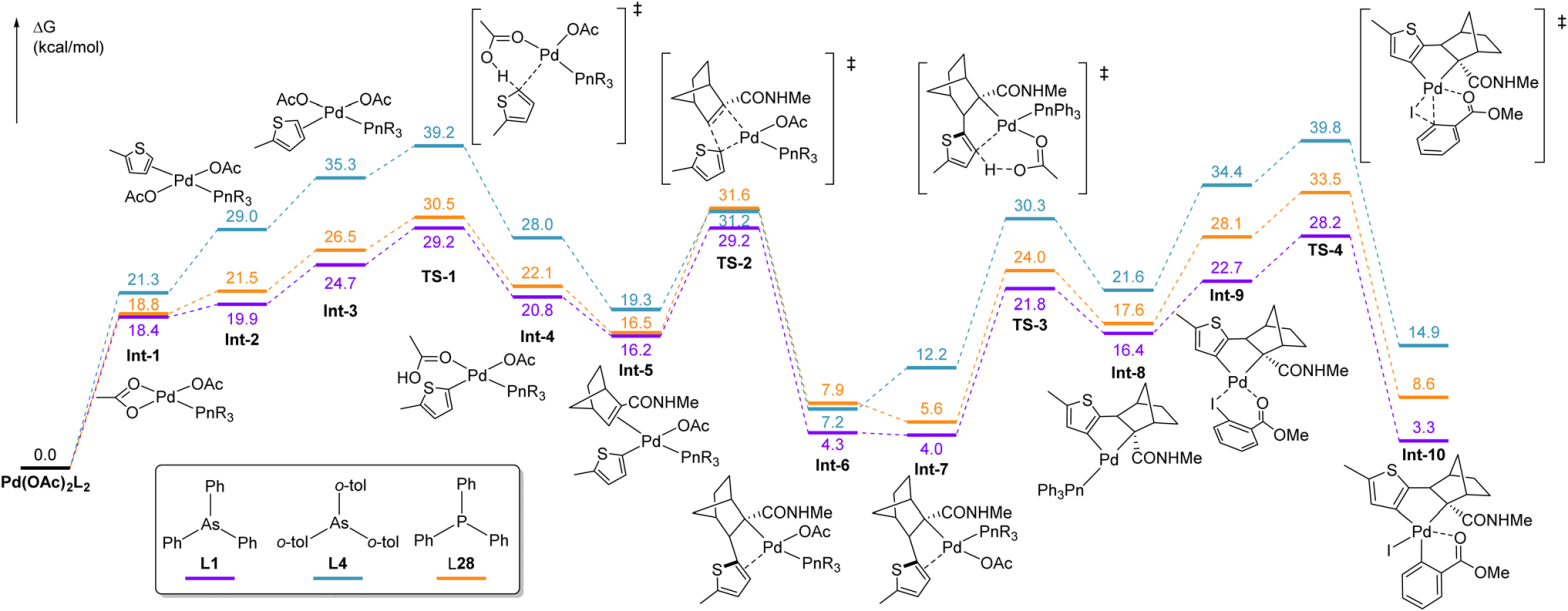
Free energy profiles of the C–H difunctionalization reaction including the first C–H palladation (TS-1), NBE insertion (TS-2), second C–H palladation (TS-3), and oxidative addition (TS-4) with L1, L4 and L28.

To gain deeper insight into the differences in ligands, the reaction pathway was analyzed using DFT calculations (Fig. 7). 2-Methylthiophene was used as the substrate for computational simplicity, and AsPh_3_ (L1), *o*-tolyl-substituted (L4) and PPh_3_ (L28) were used as representative ligands. Geometry optimizations were performed using dispersion-corrected DFT calculations at the B3LYP-D3BJ level (LANL2DZ for Pd and I, and 6-31++G(d,p) for other atoms). The Gibbs free energies at 298 K were determined by vibrational analyses (SDD for Pd and I, and 6-311G(d,p) for the other elements) at each step of the reaction. The reaction pathway is similar to that of the difunctionalization of indoles reported by Dong.^13*e*^ The initial C–H activation step (TS-1) and NBE insertion (TS-2) showed similar energy barriers with L1 (29.2 and 29.2 kcal mol^−1^, respectively) and L28 (30.5 and 31.6 kcal mol^−1^, respectively) ligands. Natural bond orbital analysis of the transition state indicated a concerted electrophilic metalation–deprotonation (eCMD) mechanism (Fig. S4).^21*b*^ This is consistent with experimental observations showing that electron-deficient thiophene bearing a methyl ester (CO_2_Me) exhibits lower reactivity (9%) than its *n*-butyl-substituted counterpart (58%).^13*c*^ The energy barriers associated with the second C–H activation (TS-3) using L1 and L28 (21.8 l and 24.0 kcal mol^−1^, respectively) were lower than those associated with the first C–H activation (TS-1) and carbo-palladation (TS-2) steps, which is consistent with the kinetic isotope effects observed in α- and β-deuterated thiophenes (*k*_H_/*k*_D_ = *α*: 1.8, *β*: 1.5). Following β-C–H activation, an aryl-NBE-palladacycle complex (Int-8) was generated, which undergoes oxidative addition with 2-iodomethylbenzoate following ligand dissociation (Int-9), forming a Pd(iv) species (Int-10) *via*TS-4 (28.2 kcal mol^−1^ for L1 and 33.5 kcal mol^−1^ for L28). Ligand dissociation at this stage is a well-known requirement in Catellani-type reactions; several studies have reported that the pathway involving ligand dissociation proceeds with a lower activation energy.^13*e*,23^ These results indicated that the activation energies were not significantly influenced by the elemental composition of the ligand; therefore, the lack of catalytic activity observed with the phosphine ligands must have another explanation. We hypothesize that phosphines are prone to oxidation after β-palladation (Int-9), rendering them catalytically inactive and thus halting the reaction at that step. This hypothesis was previously suggested by Dong, and the catalytic activity observed using L26 and L29 showed similar profiles under ligandless conditions; however, no experimental evidence for this has yet been reported.^13*e*^ In contrast, free arsine ligands remain active during this stage owing to their superior resistance to oxidation, thereby facilitating the smooth progression of the catalytic cycle.

**Fig. 8:**
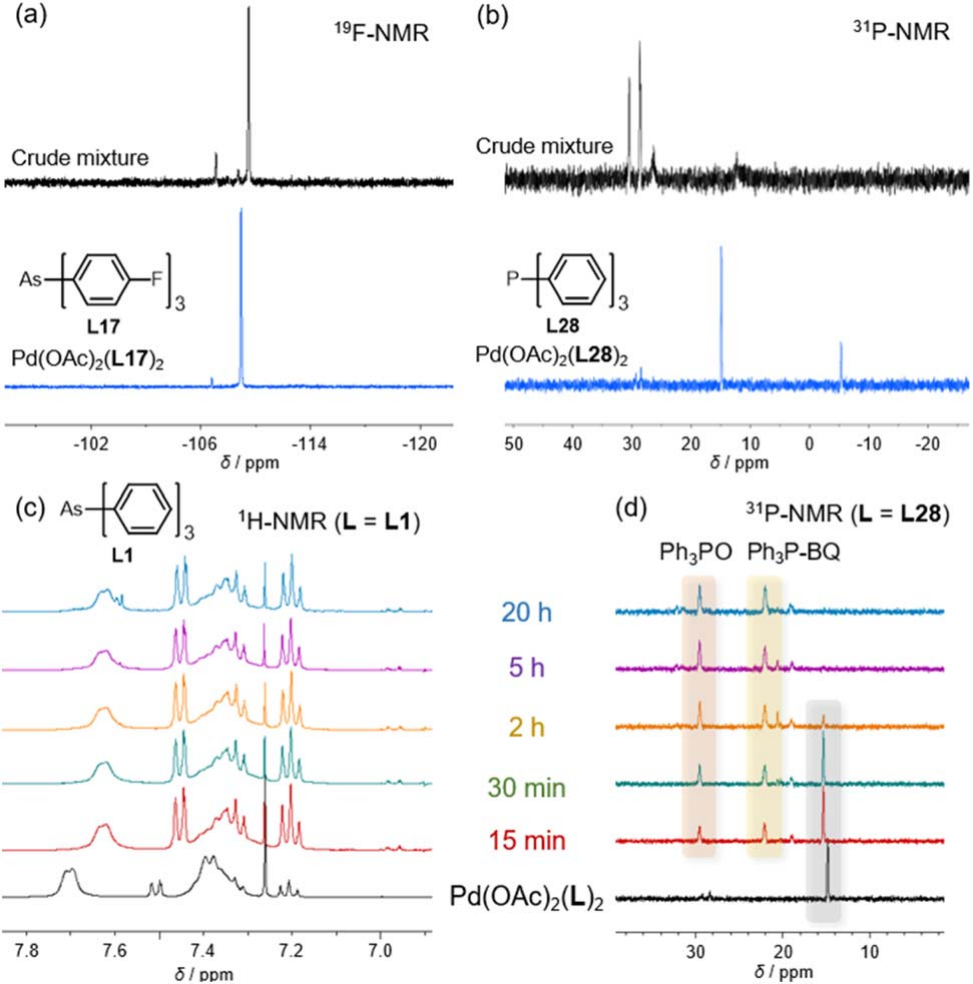
NMR spectra of the Pd(OAc) complexes and reaction mixtures after the catalytic reactions using (a) L17 (^19^F-NMR, 376 MHz) and (b) L28 (^31^P-NMR, 162 MHz) in EtOAc. Trace of the mixture of (c) L1 (^1^H-NMR, 400 MHz) and (d) L28 (^31^P-NMR, 162 MHz) (0.2 eq.), BQ (1 eq.), and AcOH (5 eq.) in CDCl_3_.

To confirm this hypothesis, we analyzed the *in situ* NMR spectra of the crude reaction mixtures using L17 and L28 to identify the Pd complexes formed (Fig. 8a and b). The ^19^F-NMR spectrum of the crude mixture containing L17 showed signals (−109.4 ppm) in similar regions to those observed in the spectrum of the Pd(OAc)_2_-ligand mixture, indicating that the ligand remained intact. A signal at −109.7 ppm was attributed to the Pd(ii) dichloride complexes with L17,^12*a*^ while the arsine oxide of L17 displayed a signal at −106.7 ppm. These observations confirm the presence of Pd(ii)-L17 complexes in the post-reaction mixture, demonstrating the stability of the catalyst under these conditions. In contrast, the phosphine system exhibited markedly different behavior. The ^31^P-NMR spectrum revealed multiple signals (30.3 and 29.3 ppm), including those corresponding to triphenylphosphine oxide (29.3 ppm); however, signals characteristic of Pd(ii)-phosphine complexes were absent, indicating catalyst decomposition. To gain further insight into catalyst deactivation *via* oxidation, the stability of Pd(OAc)_2_(L)_2_ was evaluated against BQ (Fig. 8c and d). Mixtures of Pd(OAc)_2_(L)_2_ (L = L1 (AsPh_3_) or L28 (PPh_3_)) and BQ in the presence of AcOH in CDCl_3_ were monitored by ^1^H-NMR. Although the arsine ligand L1 showed negligible change after 24 h, the phosphine ligand L28 was oxidized to form Ph_3_P = O and a Ph_3_P–BQ adduct after only 15 min at 25 °C, with the starting material being completely consumed after 5 h. These observations indicate that phosphines can be oxidized under these reaction conditions at 25 °C, even when coordinated to Pd, whereas arsine ligands are more resistant to oxidation. In the model reaction at 80 °C, oxidation of the phosphine likely proceeds even more rapidly.

These findings are consistent with our experimental observations and explain the inactivity of triarylphosphines L28–L36 in this reaction. Although phosphine and arsine ligands assist the reactions, phosphine ligands dissociate prior to oxidative addition, releasing phosphines that undergo oxidation or other deactivation pathways. The resulting ligand-free Pd species then catalyzes the Heck reaction between iodoarenes and olefins and the Catellani reaction between two iodoarenes and an olefin. Under ligand-free conditions, the reaction yielded the Heck product 3 (6%) and Catellani product 4 (11%). In contrast, the high oxidation resistance of arsines prevents the oxidation of the dissociated ligands, thereby allowing the catalytic cycle to proceed.

**Fig. 9:**
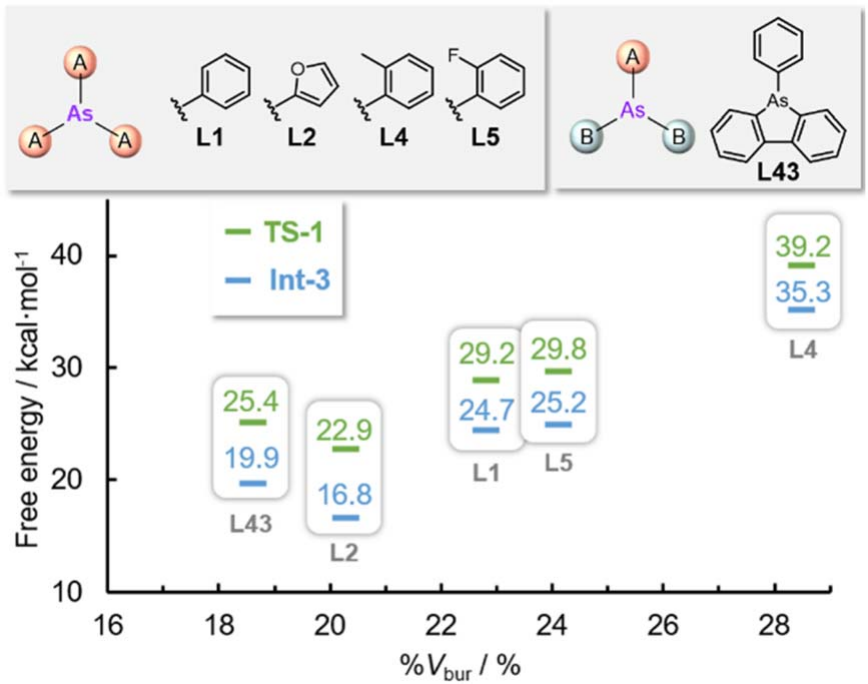
Plots of the energy profiles at Int-3 and TS-1*vs.* steric hindrance (%*V*_bur_) for L1, L2, L4, L5, and L43. The free energies are given in kcal mol^−1^.

To determine the steric influence of the arsine ligands on this reaction, the energy profiles of AsPh_3_ (L1) and *o*-tolyl-substituted L4 were compared. Using the sterically hindered arsine ligand L4, the activation energy of the first C–H activation step (39.2 kcal mol^−1^) was considerably higher than that using L1 (29.2 kcal mol^−1^). This was attributed to the steric bulk of the ligand, which inhibits the approach of thiophene toward the palladium center. Consequently, the relative energies of intermediates Int-2 and Int-3 were higher (29.0 and 35.3 kcal mol^−1^, respectively) than those obtained using L1 (21.5 and 26.5 kcal mol^−1^, respectively), which inhibits the first C–H palladation. To confirm this hypothesis, DFT calculations were performed to estimate the relative energies of Int-3 and TS-1 with various arsine ligands (L1, L2, L4, L5, and L43) having different steric properties (%*V*_bur_ = 18.6–28.5%) (Fig. 9). Although the electronic nature of the ligand also affected the activation energy, the activation energies of the formation of TS-1 increased with the steric hindrance of the ligand. The observed steric repulsions between the *o*-protons or substituents and the C4-proton of thiophene destabilized the transition state.

## Conclusions

Thirty-six arsine ligands and nine types of phosphine ligands were synthesized, and their electron-donating ability (TEP) and steric parameters (cone angle and %*V*_bur_) were evaluated using a computational approach. These ligands were applied in the Pd-catalyzed C–H difunctionalization of thiophene, a reaction that proceeds efficiently with triphenylarsine but fails with conventional phosphine ligands. The structure–activity relationship revealed that the catalytic reaction requires arsine ligands with TEP values ranging from 2066 to 2075 cm^−1^ and %*V*_bur_ values between 21.5% and 23.5%, whereas the cone angle showed no clear correlation with the catalytic activity. Additionally, the oxidative stability of trivalent arsenic is a key factor affecting the catalyst stability, as phosphines are susceptible to oxidation under reaction conditions owing to the high oxophilicity of phosphorus. These results highlight the potential utility of arsine ligands in oxidative coupling reactions, in which their inherent oxidative stability offers a distinct advantage over conventional phosphine ligands. These findings strongly support the hypothesis that arsine ligands can significantly broaden the chemical space accessible *via* transition metal catalysis. Ongoing studies are exploring the use of arsine-ligated transition-metal complexes in novel transformations that remain inaccessible using conventional phosphine ligands.

## Author contributions

A. Sumida: synthesis, structural analysis, data curation, writing – original draft; K. Yamamoto: synthesis, structural analysis, data curation, writing – original draft; T. Iwamoto: conceptualization, investigation, writing – review and editing, supervision; K. Naka: conceptualization, investigation, writing – review and editing, supervision; H. Imoto: conceptualization, investigation, writing – original draft, writing – review and editing, funding acquisition project administration, supervision.

## Conflicts of interest

There are no conflicts to declare.

## Supplementary Material

SC-016-D5SC05285H-s001

SC-016-D5SC05285H-s002

## Data Availability

The data supporting this article have been included as part of the supplementary information (SI). Supplementary information: experimental detail, NMR spectra, computational calculations. See DOI: https://doi.org/10.1039/d5sc05285h.
