# Supplementary material for: Structural effects of arsine ligands on C–H difunctionalization of thiophene
Source: Chem Sci. 2025 Oct 10;16(44):20843–50. doi: 10.1039/d5sc05285h (PMC12529081; doi:10.1039/d5sc05285h)
Supplement: SC-016-D5SC05285H-s001 [file SC-016-D5SC05285H-s001.pdf]

Supplementary Information (SI) for Chemical Science.
This journal is © The Royal Society of Chemistry 2025

# Structural Effects of Arsine Ligands on C–H Difunctionalization of Thiophene

Akifumi Sumida,[a] Kaisei Yamamoto,[a] Takahiro Iwamoto,[a] Kensuke Naka,[ab] and Hiroaki Imoto*[abc]

*[a] Dr. A. Sumida, K. Yamamoto, Dr. T. Iwamoto, Prof. Dr. K. Naka, Prof. Dr. H. Imoto*
*Faculty of Molecular Chemistry and Engineering, Kyoto Institute of Technology,*
*Goshokaido-cho, Matsugasaki, Sakyo-ku, Kyoto 606-0962, Japan*
*E-mail: himoto@kit.ac.jp (HI)*
*Homepage: http://www.cis.kit.ac.jp/~kenaka/index_eng.html*

*[b] Materials Innovation Lab, Kyoto Institute of Technology,*
*Goshokaido-cho, Matsugasaki, Sakyo-ku, Kyoto 606-0962, Japan.*

*[c] FOREST, Japan Science and Technology Corporation (JST)*
*Honcho 4-1-8, Kawaguchi, Saitama 332-0012, Japan.*

**Contents:**

1 General Information
2 Materials
3 Synthesis
4 Electronic/steric parameters and results of the ligand screening
5 Kinetic study
6 Computational details
7 NMR spectra
8 References

## 1. General Information

All reactions were carried under dry nitrogen atmosphere otherwise noted. Unless otherwise noted, all commercial reagents were used without additional purification. All dry solvents were stored with molecular sieves. $^1H$ (400 MHz), $^{13}C\{^1H\}$ (100 MHz), $^{19}F$ (376 MHz), and $^{31}P$ (162 MHz) NMR spectra were recorded on a Bruker AVANCE III 400 NMR spectrometer using $Me_4Si$ (TMS) as an internal standard. The following abbreviations are used; s: singlet, d: doublet, t: triplet, q: quartet, m: multiplet. High-resolution mass spectra (HRMS) were obtained on a JEOL JMS-SX102A spectrometer.

## 2. Materials.

Palladium(II) acetate ($Pd(OAc)_2$), triphenylarsine (**L1**), methyl 2-iodebezoate, diisobutylaluminium hydride (DIBAL-H, 1.0 M in toluene), 1,3,5-triaza-7-phosphaadamantane (**L35**), and trimethylolpropane phosphite (**L36)** were purchased from Sigma Aldrich. Dichloromethane ($CH_2Cl_2$), ethyl acetate (EtOAc), ethanol (EtOH), methanol (MeOH), concentrated hydrochloric acid ($HCl_{aq.}$), acetic acid (AcOH), sodium sulfate ($Na_2SO_4$), magnesium, methyl acrylate, and furan were purchased from Nacalai Tesque, Inc. Anhydrous solvents, diethyl ether ($Et_2O$), *n*-butyllithium (*n*-BuLi, 1.6 M hexane solution) hexane, ammonium chloride ($NH_4Cl$), deuterium oxide ($D_2O$), palladium-activated carbon (Pd 10%), triphenylphosphine (**L28**), triphenylphosphate (**L31**), and tris(4-chlorophenyl)phosphine (**L32**) were purchased from FUJIFILM Wako Pure Chemical Industry, Ltd. 1,3,5-Trimethoxybenzene, 1,4-benzoquinone (BQ), 5-bromo-*m*-xylene, 2-bromofluorobenzene, 1-bromo-3,5-difulorobenzene, 1-bromo-3-chlorobenzene, 1-bromo-3,5-*tert*-butylbenzene, 4-bromotoluene, ethynylbenzene, 2-bromobiphenyl, tri(2-furyl)phosphine (**L29**), tri(*o*-tolyl)phosphine (**L30**), and trimethylphosphine (**L34**) were purchased from Tokyo Chemical Industry Co., Ltd. Tetrahydrofuran (THF) anhydrous and $Et_2O$ anhydrous were purchased from Kanto Chemical Co., Inc. 2-Butylthiophene,[1] 4-bromo-2-butylthiophene,[2] bicyclo[2.2.1]hepta-2,5-diene-2-carbox2ylic acid,[3] *N*-methylbicyclo[2,2,1]hept-2-ene-2-carboxamide (NBE),[4] *cyclo*-$(AsPh)_6$,[5] $AsBr_3$,[6] $AsPh_2I$,[7] tris(2-methylphenyl)arsine (**L4**)[8], tris[2-(trifluoromethyl)phenyl]arsine (**L6**),[8] tris(2-methoxyphenyl)arsine (**L7**),[8] tris[3,5-di(trifluoromethyl)phenyl]arsine (**L14**),[8] tris(3,5-dimethoxyphenyl)arsine (**L15**),[8] tris(4-methylphenyl)arsine (**L16**),[8] tris(4-fluorophenyl)arsine (**L17**),[8] tris[4-(trifluoromethyl)phenyl]arsine (**L18**),[8] tris(4-methoxyphenyl)arsine (**L19**),[8] tris[4-(dimethylamino)phenyl]arsine (**L20**),[9] 2-(dicyclohexylarsino)biphenyl (**L21**),[10] 2-(dicyclohexylarsino)-2'-methylbiphenyl (**L22**),[10] 2-(dicyclohexylarsino)-2'-(dimethylamino)biphenyl (**L23**),[10] 2-(dicyclohexylarsino)-2',6'-dimethoxy-1,1'-biphenyl (**L24**),[10] 2-(dicyclohexylarsino)-2',6'-diisopropoxybiphenyl (**L25**),[10] 2-(dicyclohexylarsino)-

2’,4’,6’-tri(isopropyl)biphenyl (**L26**),[10] 2-(dicyclohexylarsino)-3,6-dimethoxy-2’,4’,6’-triisopropyl-1,1’-biphenyl (**L27**),[10] 9-phenylarsafulorene (**L43**),[11] 10-phenyl-10H-phenoxarsine (**L44**),[12] and benzoxarsorino[2,3,4-kl] phenoxarsine (**L45**)[13] were prepared according to literature procedures.

## 3. Synthesis

*Synthesis of 2-bromo-1,3-benzodithiaarsole*

To a suspension of *o*-benzenedithiol (18.1 g, 127 mmol) and $As_2O_3$ (12.9 g, 65.2 mmol), HBr *aq.* (110 mL) was added, and the mixture was heated at 100 °C for 2 h. The reaction mixture was cooled to room temperature and the solvent was removed by the decantation. The residue was triturated with MeOH followed by ether to give **1** as a yellow solid (34.2 g, 91%). Identification was carried out by comparing the results of the $^1$H-NMR measurements with the literature.[10]

*The general procedure A for arsine ligand ($AsR_3$ type)*

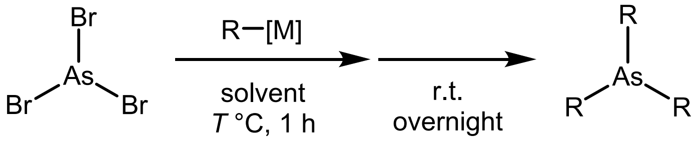


To a suspension of arylbromide (3.2 eq.) in anhydrous ethereal solvent at low temperature, *n*-BuLi in hexane (1.6 M, 3.3 eq.) was added dropwise and the resulting mixture was stirred for 1h. To this solution was added dropwise a solution of $AsBr_3$ (1.0 eq.) in anhydrous ethereal solvent. The reaction mixture was warmed to ambient temperature and stirred overnight. Then, the reaction was quenched with concentrated $NH_4Cl_{aq}$, and the aqueous layer was extracted with $Et_2O$. The combined organic layer was dried over $Na_2SO_4$ and filtered. The volatiles were removed *in vacuo* and the residue was purified with $SiO_2$ chromatography or by recrystallization to give the title compounds.

*The general procedure B for arsine ligand ($AsR_3$ type)*

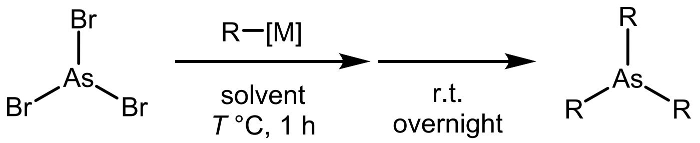


To a suspension of arylbromide (3.2 eq.) and magnesium (3.3 eq.) in anhydrous THF at 0 °C, a toluene solution of DIBAL-H (1.0 M, 2 drops) was added, and the resulting mixture was stirred for 1 h. To this solution was added dropwise a solution of $AsBr_3$ (1.0 eq.) in anhydrous

THF at 0 °C. The reaction mixture was warmed to ambient temperature and stirred overnight. Then, the reaction was quenched with concentrated $NH_4Cl_{aq}$, and the aqueous layer was extracted with $Et_2O$. The combined organic layer was dried over $Na_2SO_4$ and filtered. The volatiles were removed *in vacuo* and the residue was purified with $SiO_2$ chromatography or by recrystallization to give the title compounds.

*Tris(2-furyl)arsine* (**L2**)

Prepared according to general procedure A: $AsBr_3$ (1.00 g, 3.18 mmol), furan (1.49 g, 21.9 mmol), anhydrous $Et_2O$ (22 mL), and *n*-BuLi (1.6 M, 9.9 mL, 16 mmol) were employed. Purified with $SiO_2$ chromatography (hexane/EtOAc) to give the title compound as colorless solid (0.465 g, 1.69 mmol, 53%). Identification was carried out by comparing the results of the $^1$H-NMR measurements with the literature.[14]

*Tris(phenylethynyl)arsine* (**L3**)

Prepared according to general procedure A: $AsBr_3$ (2.00 g, 6.36 mmol), ethynylbenzene (2.27 g, 22.2 mmol), anhydrous $Et_2O$ (90 mL), and *n*-BuLi (1.6 M, 14 mL, 23 mmol) were employed. Recrystallization from $CH_2Cl_2$/MeOH to give the title compound as colorless solid (1.82 g, 4.80 mmol, 76%). $^1$H-NMR ($CDCl_3$, 400 MHz): $\delta$ = 7.57–7.54 (m, 6H), 7.38–7.31 (m, 9H) ppm. $^{13}$C{$^1$H}-NMR ($CDCl_3$, 100 MHz): $\delta$ = 132.2, 129.2, 128.3, 122.3, 103.9, 81.2 ppm. HR-FAB-MS (m/z): calculated for $C_{24}H_{16}As^+$ $[M+H]^+$; 379.0462, observed; 379.0464.

*Tris(2-fluorophenyl)arsine* (**L5**)

Prepared according to general procedure A: $AsBr_3$ (1.81 g, 5.75 mmol), 2-bromofluorobenzene (3.22 g, 18.4 mmol), anhydrous THF (33 mL), and *n*-BuLi (1.6 M, 12 mL, 19 mmol) were employed. Purified with $SiO_2$ chromatography (hexane/EtOAc) to give the title compound as colorless solid (0.918 g, 2.55 mmol, 44%). $^1$H-NMR ($CDCl_3$, 400 MHz): $\delta$ = 7.40–7.34 (m, 1H), 7.11–7.06 (m, 2H), 6.94–6.91 (m, 1H) ppm. $^{13}$C{$^1$H}-NMR ($CDCl_3$, 100 MHz): $\delta$ = 163.5 (d, $J$ = 242 Hz), 134.6 (d, $J$ = 6.0 Hz), 131.4 (d, $J$ = 8.0 Hz), 124.9 (d, $J$ = 3.0 Hz), 123.1 (d, $J$ = 25 Hz), 115.2 (d, $J$ = 24 Hz) ppm. $^{19}$F-NMR ($CDCl_3$, 376 MHz): $\delta$ = −103.2 (quartette, $J$ = 8.2 Hz, 1F) ppm. HR-FAB-MS (m/z): calculated for $C_{18}H_{13}F_3As$ $[M+H]^+$; 361.0180, observed; 361.0196.

*Tris(4-methylphenyll)arsine* (**L8**)

Prepared according to general procedure B: $AsBr_3$ (1.00 g, 3.17 mmol), 3-bromotoluene (1.69 g, 10.2 mmol), anhydrous THF (10 mL), magnesium (255 mg, 10.5 mmol), and DIBAL-H (1.0 M, 2 drops) were employed. Recrystallization from $CH_2Cl_2$/MeOH to give the title

compound as colorless solid (0.818 g, 2.35 mmol, 74%). $^1$H-NMR ($CDCl_3$, 400 MHz): $\delta$ = 7.22–7.18 (m, 6H), 7.13–7.08 (m, 3H), 2.29 (s, 9H) ppm. $^{13}$C{1H}-NMR ($CDCl_3$, 100 MHz): $\delta$ = 139.6, 138.1, 134.4, 130.7, 129.2, 128.4, 21.4 ppm. HR-FAB-MS (m/z): calculated for $C_{21}H_{21}As$ $[M]^+$; 348.0859, observed; 348.0859.

*Tris(3-chlorophenyl)arsine* (**L9**)

Prepared according to general procedure A: $AsBr_3$ (1.00 g, 3.18 mmol), 1-bromo-3-chlorobenzene (2.12 g, 11.0 mmol), anhydrous $Et_2O$ (22 mL), and *n*-BuLi (1.6 M, 7.2 mL, 11 mmol) were employed. Purified with $SiO_2$ chromatography (hexane) to give the title compound as colorless oil (0.788 g, 1.92 mmol, 60%). $^1$H-NMR ($CDCl_3$, 400 MHz): $\delta$ = 7.36–7.27 (m, 9H), 7.17 (dt, $J$ = 7.3, 1.3 Hz, 3H) ppm. $^{13}$C{$^1$H}-NMR ($CDCl_3$, 100 MHz): $\delta$ = 140.5, 135.1, 133.2, 131.6, 130.1, 129.2 ppm. HR-FAB-MS (m/z): calculated for $C_{18}H_{12}Cl_3As$ $[M]^+$; 407.9221, observed; 407.9232.

*Tris(3,5-dimethylphenyl)arsine* (**L10**)

Prepared according to general procedure A: $AsBr_3$ (1.50 g, 4.77 mmol), 5-bromo-*m*-xylene (3.08 g, 16.7 mmol), anhydrous THF (33 mL), and *n*-BuLi (1.6 M, 11 mL, 17 mmol) were employed. Recrystallization from $CH_2Cl_2$/EtOH to give the title compound as colorless solid (0.946 g, 2.42 mmol, 51%). $^1$H-NMR ($CDCl_3$, 400 MHz): $\delta$ = 6.96 (s, 6H), 6.95 (s, 3H), 2.26 (s, 18H) ppm. $^{13}$C{$^1$H}-NMR ($CDCl_3$, 100 MHz): $\delta$ = 139.7, 137.9, 131.4, 130.2 ppm. HR-FAB-MS (m/z): calculated for $C_{24}H_{27}As$ $[M]^+$; 390.1329, observed; 390.1322.

*Tris(3,5-di-tert-butylphenyl)arsine* (**L11**)

Prepared according to general procedure B: $AsBr_3$ (1.00 g, 3.17 mmol), 1-bromo-3,5-*tert*-butylbenzene (1.88 g, 10.1 mmol), anhydrous THF (10 mL), magnesium (255 mg, 10.5 mmol), and DIBAL-H (1.0 M, 2 drops) were employed. Recrystallization from $CH_2Cl_2$/MeOH to give the title compound as colorless solid (1.07 g, 2.26 mmol, 71%). $^1$H-NMR ($CDCl_3$, 400 MHz): $\delta$ = 6.96 (s, 6H), 6.95 (s, 3H), 2.26 (s, 18H) ppm. $^{13}$C{$^1$H}-NMR ($CDCl_3$, 100 MHz): $\delta$ = 150.5, 139.4, 127.9, 122.0, 34.9, 31.4 ppm. HR-FAB-MS (m/z): calculated for $C_{42}H_{63}As$ $[M]^+$; 642.4146, observed; 642.4147.

*Tris(m-terphenyl)arsine* (**L12**)

Prepared according to general procedure A: $AsBr_3$ (1.00 g, 3.18 mmol), 5-bromo-*m*-terphenyl (3.14 g, 10.2 mmol), anhydrous THF (33 mL), and *n*-BuLi (1.6 M, 6.6 mL, 10 mmol) were employed. Recrystallization from $CH_2Cl_2$/hexane to give the title compound as colorless solid (1.00 g, 1.31 mmol, 41%). $^1$H-NMR ($CDCl_3$, 400 MHz): $\delta$ = 7.79 (t, $J$ = 1.6 Hz, 3H), 7.76

(d, $J$ = 1.6 Hz, 6H), 7.58–7.55 (m, 12H), 7.40–7.37 (m, 12H), 7.33–7.30 (m, 6H) ppm. $^{13}C\{^1H\}$-NMR ($CDCl_3$, 100 MHz): $\delta$ = 142.1, 140.9, 140.7, 131.5, 128.8, 127.5, 127.3, 126.7 ppm. HR-FAB-MS (m/z): calculated for $C_{54}H_{39}As$ $[M]^+$; 762.2268, observed; 762.2260.

*Tris(3,5-difluorophenyl)arsine* (**L13**)

Prepared according to general procedure B: $AsBr_3$ (1.00 g, 3.17 mmol), 1-bromo-3,5-difulorobenzene (1.96 g, 10.5 mmol), anhydrous THF (10 mL), magnesium (253 mg, 10.1 mmol), and DIBAL-H (1.0 M, 2 drops) were employed. Purified with $SiO_2$ chromatography (hexane) to give the title compound as colorless solid (0.522 g, 1.26 mmol, 40%).$^1H$-NMR ($CDCl_3$, 400 MHz): $\delta$ = 6.88–6.79 (m, 9H) ppm. $^{13}C\{^1H\}$-NMR ($CDCl_3$, 100 MHz): $\delta$ = 163.3 (dd, $J$ = 12, 11 Hz), 141.3 (t, $J$ = 6.0 Hz), 116.1 (dd, $J$ = 11, 7.0 Hz), 105.3 (t, $J$ = 25.0 Hz) ppm. $^{19}F$-NMR ($CDCl_3$, 376 MHz): $\delta$ = −108.8 (t, $J$ = 7.5 Hz, 1F) ppm. HR-FAB-MS (m/z): calculated for $C_{18}H_9AsF_6$ $[M]^+$; 413.9824, observed; 413.9814.

*The general procedure for arsine ligand ($AsPhR_2$ type)*

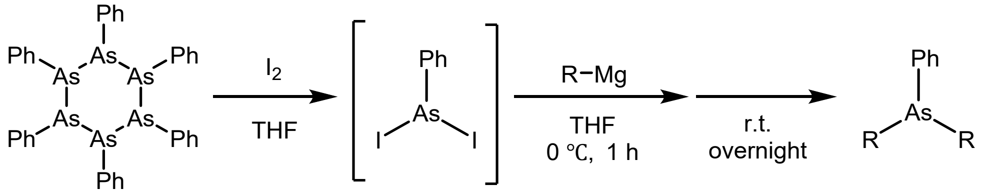


To a suspension of arylbromide (12.6 eq.) and magnesium (13.2 eq.) in anhydrous THF at 0 °C, a toluene solution of DIBAL-H (1.0 M, 2 drops) was added and the resulting mixture was stirred for 1 h. To this solution was added dropwise a solution of *cyclo*-$(AsPh)_6$ (1.0 eq.) and $I_2$ (6.0 eq.) in anhydrous THF at 0 °C. The reaction mixture was warmed to ambient temperature and stirred overnight. Then, the reaction was quenched with concentrated $NH_4Cl_{aq}$, and the aqueous layer was extracted with $Et_2O$. The combined organic layer was dried over $Na_2SO_4$ and filtered. The volatiles were removed *in vacuo* and the residue was purified with $SiO_2$ chromatography or by recrystallization to give the title compounds.

*Bis(4-methylphenyl)phenylarsine* (**L37**)

Prepared according to general procedure: *cyclo*-$(AsPh)_6$ (0.503 g, 0.551 mmol), $I_2$ (0.836 g, 3.29 mmol), 4-bromotoluene (1.19 g, 6.95 mmol), anhydrous THF (14 mL), magnesium (177 mg, 7.28 mmol), and DIBAL-H (1.0 M, 2 drops) were employed. Recrystallization from $CH_2Cl_2$/MeOH to give the title compound as colorless solid (0.804 g, 2.41 mmol, 73%). $^1H$-NMR ($CDCl_3$, 400 MHz): $\delta$ = 7.34–7.28 (m, 5H), 7.23–7.21 (m, 4H), 7.14–7.12 (m, 4H) ppm.

$^{13}C\{^1H\}$-NMR ($CDCl_3$, 100 MHz): $\delta$ = 140.2, 138.2, 136.2, 133.7, 133.6, 129.4, 128.6, 128.2, 21.3 ppm. HR-FAB-MS (m/z): calculated for $C_{20}H_{19}As$ $[M]^+$; 334.0703, observed; 334.0700.

*Bis(3,5-dimethylphenyl)phenylarsine* (**L38**)

Prepared according to general procedure: *cyclo*-$(AsPh)_6$ (0.497 g, 0.545 mmol), $I_2$ (0.828 g, 3.26 mmol), 5-bromo-*m*-xylene (1.27 g, 6.86 mmol), anhydrous THF (14 mL), magnesium (175 mg, 7.20 mmol), and DIBAL-H (1.0 M, 2 drops) were employed. Purified with $SiO_2$ chromatography (hexane) to give the title compound as colorless solid (0.751 g, 2.07 mmol, 63%). $^1H$-NMR ($CDCl_3$, 400 MHz): $\delta$ = 7.35–7.30 (m, 5H), 6.96 (s, 6H), 2.25 (s, 12H) ppm. $^{13}C\{^1H\}$-NMR ($CDCl_3$, 100 MHz): $\delta$ = 140.1, 139.4, 138.0, 133.7, 131.4, 130.22, 128.5, 128.2, 21.3 ppm. HR-FAB-MS (m/z): calculated for $C_{22}H_{23}As$ $[M]^+$; 362.1016, observed; 362.1022.

*Bis(3,5-di-tert-butylphenyl)phenylarsine* (**L39**)

Prepared according to general procedure: *cyclo*-$(AsPh)_6$ (0.507 g, 0.556 mmol), $I_2$ (0.846 g, 3.33 mmol), 1-bromo-3,5-*tert*-butylbenzene (1.86 g, 6.91 mmol), anhydrous THF (14 mL), magnesium (176 mg, 7.24 mmol), and DIBAL-H (1.0 M, 2 drops) were employed. Recrystallization from $CH_2Cl_2$/EtOH to give the title compound as colorless solid (1.19 g, 2.85 mmol, 86%). $^1H$-NMR ($CDCl_3$, 400 MHz): $\delta$ = 7.37–7.28 (m, 7H), 7.16 (d, *J* = 1.8 Hz, 4H), 1.23 (s, 36H) ppm. $^{13}C\{^1H\}$-NMR ($CDCl_3$, 100 MHz): $\delta$ = 150.5, 140.7, 138.8, 133.7, 128.3, 128.0, 127.9, 122.2, 34.9, 31.4 ppm. HR-FAB-MS (m/z): calculated for $C_{34}H_{47}As$ $[M]^+$; 530.2894, observed; 530.2892.

*The general procedure for arsine ligand ($AsPh_2R$ type)*

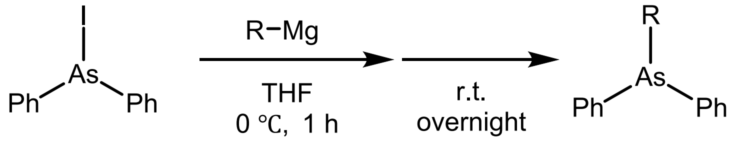


To a suspension of arylbromide (1.05 eq.) and magnesium (1.1 eq.) in anhydrous THF at 0 °C, a toluene solution of DIBAL-H (1.0 M, 2 drops) was added and the resulting mixture was stirred for 1 h. To this solution was added dropwise a solution of $AsPh_2I$ (1.0 eq.) in anhydrous THF at 0 °C. The reaction mixture was warmed to ambient temperature and stirred overnight. Then, the reaction was quenched with concentrated $NH_4Cl_{aq}$, and the aqueous layer was extracted with $Et_2O$. The combined organic layer was dried over $Na_2SO_4$ and filtered. The volatiles were removed *in vacuo* and the residue was purified with $SiO_2$ chromatography or by recrystallization to give the title compounds.

*(3,5-dimethylphenyl)diphenylarsine* (**L40**)

Prepared according to general procedure: $AsPh_2I$ (1.18 g, 3.32 mmol), 5-bromo-*m*-xylene (0.635 g, 3.48 mmol), anhydrous THF (10 mL), magnesium (86.9 mg, 3.65 mmol), and DIBAL-H (1.0 M, 2 drops) were employed. Purified with $SiO_2$ chromatography (hexane) to give the title compound as colorless solid (0.753 g, 2.25 mmol, 68%). $^1$H-NMR ($CDCl_3$, 400 MHz): $\delta$ = 7.35–7.30 (m, 10H), 6.96 (s, 1H), 6.95 (s, 2H), 2.25 (s, 12H) ppm. $^{13}$C{$^1$H}-NMR ($CDCl_3$, 100 MHz): $\delta$ = 139.9, 139.2, 138.0, 133.7, 131.4, 130.3, 128.6, 128.3, 21.3 ppm. HR-FAB-MS (m/z): calculated for $C_{20}H_{19}As$ $[M]^+$; 334.0703, observed; 334.0694.

*(3,5-Di-tert-butylphenyl)diphenylarsine* (**L41**)

Prepared according to general procedure: $AsPh_2I$ (1.02 g, 2.87 mmol), 1-bromo-3,5-*tert*-butylbenzene (0.795 g, 3.01 mmol), anhydrous THF (8.6 mL), magnesium (74.6 mg, 3.15 mmol), and DIBAL-H (1.0 M, 2 drops) were employed. Recrystallization from $CH_2Cl_2$/EtOH to give the title compound as colorless solid (0.841 g, 2.01 mmol, 70%). $^1$H-NMR ($CDCl_3$, 400 MHz): $\delta$ = 7.37–7.29 (m, 11H), 7.18 (d, $J$ = 1.8 Hz, 2H), 1.24 (s, 18H) ppm. $^{13}$C{$^1$H}-NMR ($CDCl_3$, 100 MHz): $\delta$ = 150.7, 140.2, 138.2, 133.7, 128.5, 128.3, 128.0, 122.3, 34.9, 31.4 ppm. HR-FAB-MS (m/z): calculated for $C_{26}H_{31}As$ $[M]^+$; 418.1642, observed; 418.1632.

*2-(Diphenylarsino)biphenyl* (**L42**)

Prepared according to general procedure: $AsPh_2I$ (1.01 g, 2.84 mmol), 2-bromobiphenyl (0.686 g, 2.98 mmol), anhydrous THF (16 mL), magnesium (75.4 mg, 3.12 mmol), and DIBAL-H (1.0 M, 2 drops) were employed. Recrystallization from $CH_2Cl_2$/EtOH to give the title compound as colorless solid (0.864 g, 2.27 mmol, 80%). Identification was carried out by comparing the results of the $^1$H NMR measurements with the literature[15].

*The general procedure for ligand screening for the Pd-catalyzed difunctionalization of thiophene*

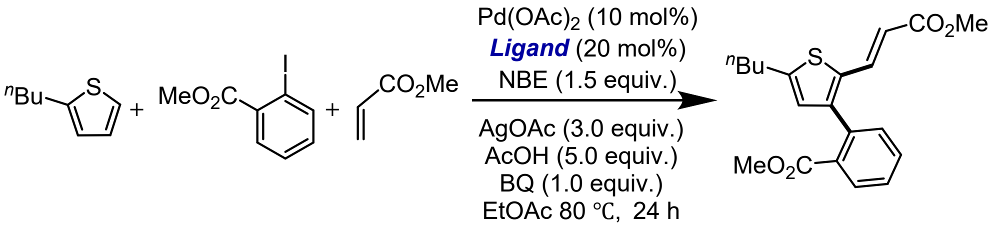


To a mixture of 2-butylthiophene (44 μL, 0.30 mmol, 1.5 eq.), 2-iodebenzoate (30 μL, 0.20 mmol, 1.0 eq.), methyl acrylate (32 μL , 0.36 mmol, 1.8 eq.), AgOAc (100 mg, 0.60 mmol, 3.0 eq.), HOAc (57 μL, 1.0 mmol, 5.0 eq.), BQ (21.6 mg, 0.2 mmol, 1.0 eq.), NBE (45.4 mg, 0.30

mmol, 1.5 eq.), $Pd(OAc)_2$ (4.6 mg, 0.02 mmol, 10 mol%), and $AsPh_3$ (12.1 mg, 0.04 mmol, 20 mol%), degassed EtOAc (1.0 mL) was added and stirred at 80 °C for 24 h under nitrogen atmosphere. The crude product wad passed through the pad of silica and 1,3,5-trimethoxybenzene (20.0 mg, 0.119 mmol) was added as an internal standard. The yield of the target compound was evaluated by $^1H$-NMR. The residue was purified by silica column chromatography to give the target compound.

*Methyl 2-[5-butyl-2-[(1E)-3-methoxy-3-oxo-1-propen-1-yl]-3-thienyl]benzoate*

Following general procedure. The residue was purified by silica column chromatography (hexane/EtOAc) to give the target compound as yellow oil (39.4 mg, 0.011 mmol, 54%). $^1H$-NMR ($CDCl_3$, 400 MHz): $\delta$ = 7.94 (dd, J = 7.7, 1.3 Hz, 1H), 7.54 (td, J = 7.5, 1.4 Hz, 1H), 7.47 – 7.43 (m, 2H), 7.26 (dd, J = 7.6, 1.2 Hz, 1H), 6.64 (s, 1H), 6.09 (d, J = 15.6 Hz, 1H), 3.70 (s, 3H), 3.68 (s, 3H), 2.86–2.77 (m, 2H), 1.72–1.65 (m, 2H), 1.42 (sextet, *J* = 7.5 Hz, 2H), 0.95 (t, J = 7.4 Hz, 3H) ppm. $^{13}C\{^1H\}$-NMR ($CDCl_3$, 100 MHz): $\delta$ = 167.7, 167.4, 147.6, 145.0, 136.5, 136.0, 132.4, 131.6, 131.6, 131.1, 130.4, 128.0, 127.8, 115.3, 52.1, 51.5, 33.4, 30.1, 22.1, 13.8 ppm. These data are in good agreement with the following literature[16].

<u>*Deuteration of thiophene*</u>

*5-d-2-Butylthiophene*

To a suspension of 2-butylthiophene (1.90 g, 13.6 mmol) in anhydrous THF (25 mL) at −78 °C, *n*-BuLi in hexane (1.6 M, 10.1 mL, 16.3 mmol) was added dropwise and the resulting mixture was stirred for 1 h. To this solution was added $D_2O$ at −78 °C. The reaction mixture was warmed to ambient temperature and stirred for 1 h. Then, the reaction was quenched with water, and the aqueous layer was extracted with $Et_2O$. The combined organic layer was dried over $Na_2SO_4$ and filtered. The volatiles were removed *in vacuo* and the residue was purified by vacuum distillation to give the title compounds as a colorless liquid (1.48 g, 10.5 mmol, 77%). Identification was carried out by comparing the results of the $^1H$ NMR measurements with the literature[17].

*4-d-2-Butylthiophene*

To a suspension of 4-bromo-2-butylthiophene (3.22 g, 14.7 mmol) in anhydrous hexane (32 mL) at −78 °C, *n*-BuLi in hexane (1.6 M, 9.6 mL, 15.4 mmol), was added dropwise, then anhydrous THF (10 mL) was added, and the resulting mixture was stirred for 1 h. To this solution was added methanol-4*d* (0.90 mL, 22.1 mmol) at −78 °C and the resulting mixture was stirred for 1h. The reaction mixture was warmed to ambient temperature and stirred for 1 h. Then, the reaction was quenched with water, and the aqueous layer was extracted with $Et_2O$. The combined organic layer was dried over $Na_2SO_4$ and filtered. The volatiles were

removed *in vacuo* and the residue was purified by vacuum distillation to give the title compounds as a colorless liquid (1.57 g, 11.1 mmol, 76%). $^1$H-NMR ($CDCl_3$, 400 MHz): $\delta$ = 7.09 (s, 1H), 6.77 (s, 1H), 2.82 (t, $J$ = 7.9 Hz, 2H), 1.66 (quintet, $J$ = 7.7 Hz, 2H), 1.39 (sextet, $J$ = 7.5 Hz, 2H), 0.93 (t, $J$ = 7.4 Hz, 3H) ppm. $^{13}$C{$^1$H}-NMR ($CDCl_3$, 100 MHz): $\delta$ = 145.8, 126.4 (t, $J$ = 25.4 Hz, 1C), 123.8, 122.6, 33.9, 29.6, 22.2, 13.8 ppm. HR-FAB-MS (m/z): calculated for $C_8H_{11}DS$ $[M]^+$; 141.0722, observed; 141.0723.

<u>*Synthesis of the precursor of NBE*</u>

*Bicyclo[2.2.1]hept-2-ene-2-carboxylic acid*

To a suspension of bicyclo[2.2.1]hepta-2,5-diene-2-carboxylic acid (15.4 g, 112.9 mmol) and ammonium formate (7.83 g, 124.1 mmol) in anhydrous MeOH (100 mL) at room temperature, 10 % Pd-C (5 % water content, 0.630 g, 0.56 mmol, 0.5 mol%) was added at once and the resulting mixture was refluxed for 72 h. Then, the reaction was cooled to room temperature and quenched with $HCl_{aq}$, and the aqueous layer was extracted with DCM. The combined organic layer was dried over $Na_2SO_4$ and filtered. The volatiles were removed to give the title compounds as a brown liquid (13.9 g, 101 mmol, 89%). Identification was carried out by comparing the results of the $^1$H-NMR measurements with the literature[18].

## 4. Electronic/steric parameters and results of the ligand screening

**Table S1.** Electronic/steric parameters and yields of the product and by-products of each ligand.

| Entry | TEP / cm$^{-1}$ | Cone Angle /° | %$V_{bur}$ /% | **1** /% | **2** /% | **3** /% | **4** /% |
|---|---|---|---|---|---|---|---|
| **L1** | 2071.8 | 158.7 | 22.8 | 58 | 12 | 0 | 0 |
| **L2** | 2079.8 | 153.7 | 20.3 | 4 | 2 | 7 | 10 |
| **L3** | 2078.5 | 162.9 | 16.2 | 0 | 0 | 5 | 5 |
| **L4** | 2069.6 | 180.3 | 28.5 | 2 | 4 | 2 | 7 |
| **L5** | 2077.3 | 161.2 | 24.1 | 3 | 0 | 6 | 8 |
| **L6** | 2080.4 | 193.5 | 33.4 | 2 | 0 | 1 | 7 |

| | | | | | | | |
|---|---|---|---|---|---|---|---|
| **L7** | 2061.7 | 175.9 | 25.2 | 4 | 5 | 3 | 13 |
| **L8** | 2070.1 | 161.8 | 22.8 | 65 | 12 | 3 | 0 |
| **L9** | 2076.9 | 162.4 | 22.9 | 29 | 4 | 3 | 5 |
| **L10** | 2068.0 | 170.5 | 22.9 | 64 | 13 | 2 | 4 |
| **L11** | 2066.0 | 195.5 | 22.9 | 39 | 11 | 6 | 11 |
| **L12** | 2069.1 | 197.8 | 23.3 | 59 | 11 | 4 | 0 |
| **L13** | 2081.7 | 159.2 | 22.8 | 7 | 2 | 10 | 9 |
| **L14** | 2086.3 | 178.6 | 22.9 | 7 | 2 | 3 | 6 |
| **L15** | 2070.2 | 162.8 | 23.1 | 48 | 10 | 6 | 7 |
| **L16** | 2069.9 | 158.6 | 22.8 | 59 | 15 | 0 | 0 |
| **L17** | 2074.4 | 159.1 | 22.9 | 59 | 7 | 4 | 4 |
| **L18** | 2079.1 | 158.1 | 22.7 | 18 | 4 | 1 | 0 |
| **L19** | 2068.7 | 158.9 | 22.8 | 24 | 5 | 0 | 0 |
| **L20** | 2063.5 | 159.6 | 22.9 | 0 | 0 | 2 | 2 |
| **L21** | 2061.0 | 167.9 | 27.3 | 0 | 0 | 1 | 9 |
| **L22** | 2059.9 | 168.6 | 27.8 | 0 | 2 | 0 | 4 |
| **L23** | 2058.2 | 169.1 | 28.1 | 0 | 0 | 1 | 7 |
| **L24** | 2058.1 | 168.6 | 27.9 | 0 | 11 | 11 | 0 |
| **L25** | 2056.7 | 169.3 | 28.2 | 0 | 0 | 12 | 7 |
| **L26** | 2057.9 | 175.3 | 28.4 | 0 | 0 | 2 | 7 |
| **L27** | 2053.4 | 180.4 | 29.0 | 0 | 2 | 2 | 9 |

| | | | | | | | |
|---|---|---|---|---|---|---|---|
| **L28** | 2067.4 | 164.1 | 31.1 | 0 | 0 | 13 | 11 |
| **L29** | 2076.3 | 160.6 | 29.0 | 0 | 0 | 15 | 5 |
| **L30** | 2065.9 | 187.2 | 36.0 | 0 | 0 | 7 | 9 |
| **L31** | 2080.7 | 169.6 | 31.5 | 0 | 0 | 11 | 9 |
| **L32** | 2071.0 | 164.5 | 31.1 | 1 | 2 | 10 | 16 |
| **L33** | 2068.7 | 169.3 | 23.7 | 0 | 0 | 5 | 6 |
| **L34** | 2065.7 | 117.0 | 23.6 | 8 | 49 | 5 | 9 |
| **L35** | 2068.5 | 112.9 | 22.8 | 2 | 11 | 0 | 0 |
| **L36** | 2087.5 | 110.2 | 21.8 | 7 | 24 | 8 | 13 |
| **L37** | 2070.6 | 158.8 | 22.8 | 62 | 11 | 1 | 2 |
| **L38** | 2069.2 | 166.5 | 22.8 | 64 | 13 | 3 | 0 |
| **L39** | 2067.9 | 184.6 | 23.1 | 50 | 12 | 6 | 7 |
| **L40** | 2070.5 | 162.9 | 22.8 | 68 | 13 | 3 | 0 |
| **L41** | 2069.8 | 173.5 | 23.1 | 55 | 12 | 5 | 5 |
| **L42** | 2067.7 | 151.8 | 23.2 | 0 | 0 | 11 | 8 |
| **L43** | 2073.8 | 140.1 | 18.6 | 58 | 14 | 0 | 0 |
| **L44** | 2074.3 | 156.9 | 21.9 | 62 | 17 | 4 | 0 |
| **L45** | 2076.6 | 121.7 | 16.8 | 10 | 0 | 1 | 2 |

## 5. Reaction study

*Kinetic Isotope Effect (KIE) investigations*

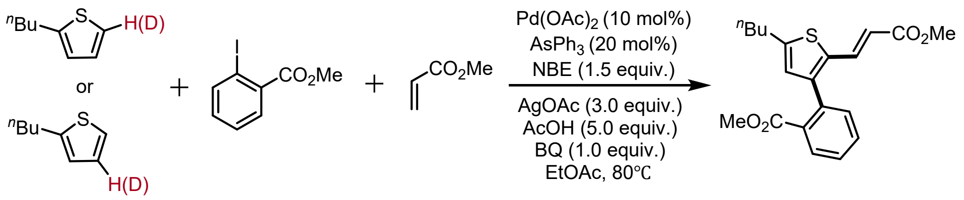


To a mixture of 2-butylthiophene (44 μL, 0.30 mmol) or 5-*d*-2-butylthiophene (44 μL, 0.30 mmol) or 4-*d*-2-butylthiophene (44 μL, 0.30 mmol), 2-iodebenzoate (30 μL, 0.20 mmol, 1.0 eq.), methyl acrylate (32 μL , 0.36 mmol, 1.8 eq.), AgOAc (100 mg, 0.60 mmol, 3.0 eq.), DOAc-$d_4$ (57 μL, 1.0 mmol, 5.0 eq.), BQ (21.6 mg, 0.2 mmol, 1.0 eq.), NBE (45.4 mg, 0.30 mmol, 1.5 eq.), $Pd(OAc)_2$ (4.6 mg, 0.02 mmol, 10 mol%), and $AsPh_3$ (12.1 mg, 0.04 mmol, 20 mol%), degassed EtOAc (1.0 mL) was added and stirred at 80 °C under nitrogen atmosphere. The reaction was followed during times, 30, 60, 90, and 120 minutes. The crude product wad passed through the pad of silica and 1,3,5-trimethoxybenzene (10.0 mg, 0.059 mmol) was added as an internal standard. The yield of the target compound was evaluated by $^1$H-NMR.

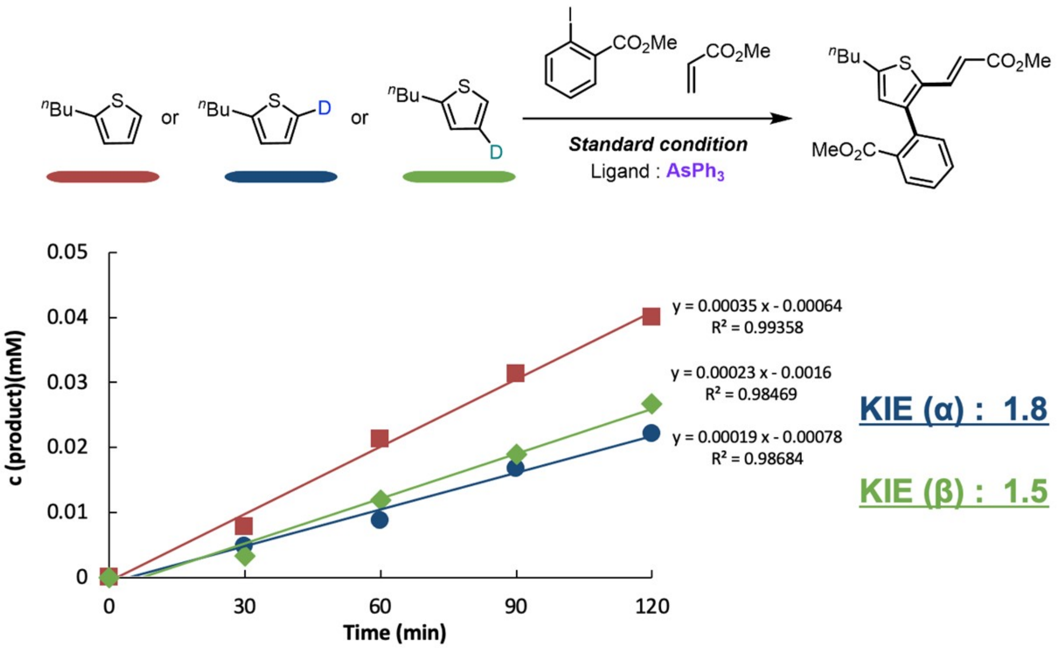


**Figure S1.** Parallel KIE of thiophene

**Table S2.** Reaction without 2-butylthiophene.

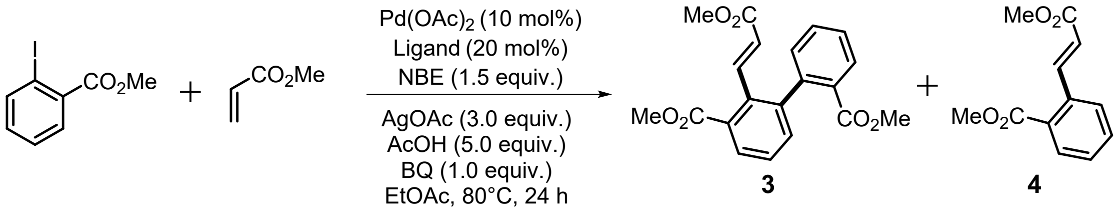


| Ligand | **3** / % | **4** / % |
|---|---|---|
| **L1** ($AsPh_3$) | 7 | trace |
| **L28** ($PPh_3$) | 17 | 8 |

**Table S3.** Reaction with 2-bromo- or iodo-methylbenzoate

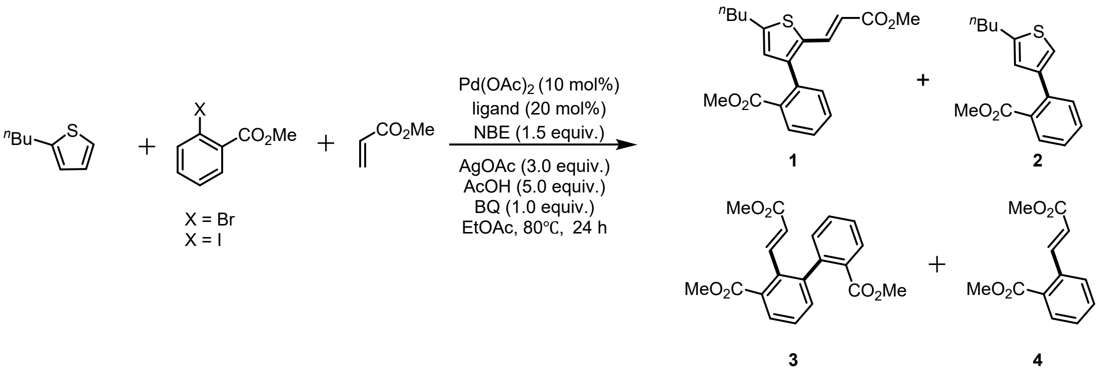


| Substrate | Ligand | **1** / % | **2** / % | **3** / % | **4** / % |
|---|---|---|---|---|---|
| X = I | **L1** ($AsPh_3$) | 58 | 12 | 0 | 0 |
| | **L28** ($PPh_3$) | 0 | 0 | 13 | 11 |
| | none | 1 | 0 | 6 | 11 |
| X = Br | **L1** ($AsPh_3$) | 9 | 2 | 0 | 6 |
| | **L28** ($PPh_3$) | 0 | 0 | 1 | 0 |
| | none | 0 | 0 | 0 | 0 |

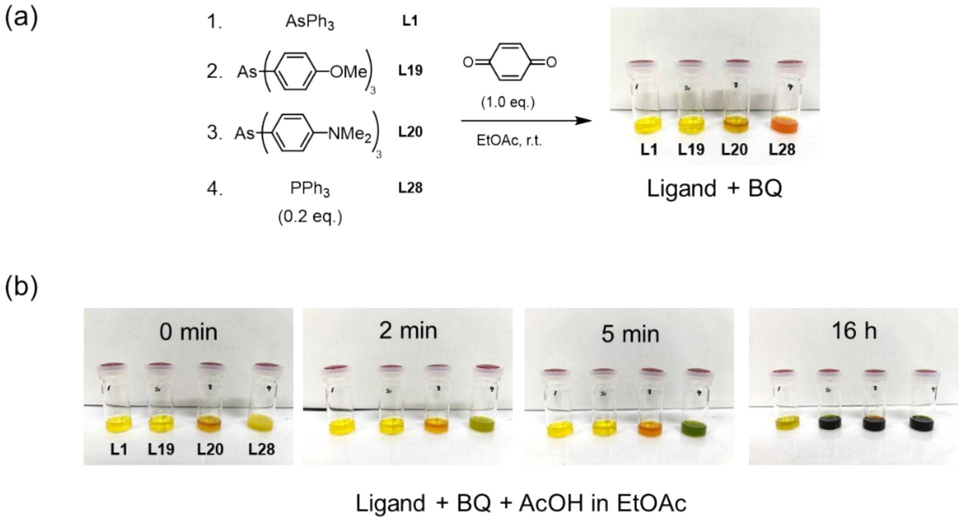


**Figure S2.** (a) The reactions between the ligand and BQ in ethyl acetate at room temperature. (b) Photographs showing the color change of the solution after the addition of AcOH.

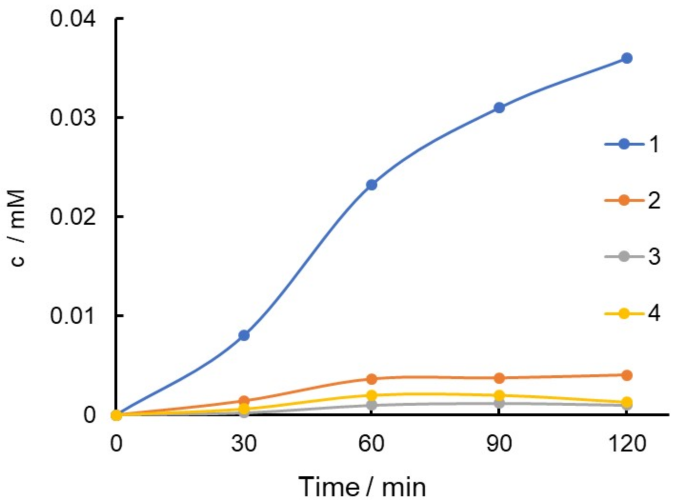


**Figure S3.** Concentration of the product **1** and by-product **2-4** using **L1** within 120 min.

## 6. **Computational details**

All density functional theory (DFT) calculations were performed using the Gaussian 16 software package. Geometries were optimized using the B3LYP functional and Grimme's D3(BJ) dispersion correction with a mixed basis set of LANL2DZ for Pd and I and 6-31G++(d,p) for other atoms. Vibrational frequencies were calculated for all the stationary points to confirm if each optimized structure is a local minimum on the respective potential energy surface or a transition state structure with only one imaginary frequency. Solvation energy corrections were calculated in EtOAc with the SMD continuum solvation model. The B3LYP functional and Grimme's D3(BJ) dispersion correction with a mixed basis set of SDD for Pd and I and 6-311G(d,p) for other atoms were used for single-point energy calculations. Natural bonding orbital (NBO) analysis was conducted by NBO 7.0 program.

Geometries and vibrational frequencies of $Ni(CO)_3$ complex were calculated using B3LYP functional and Grimme's D3 dispersion correction with a mixed basis set of LANL2DZ for Ni and def2-TZVP for other atoms. Based on the optimized geometries steric parameters were calculated by the exact cone angle program and SambVca2 software.

**Table S4.** Wiberg bond indices of Pd–$C_{ipso}$(thiophene) and H–$C_{ipso}$(thiophene) in **TS-1** structure.

| Ligand | $\%V_{bur}$ | WBI (Pd–$C_{ipso}$) | WBI (H–$C_{ipso}$) |
|---|---|---|---|
| **L1** | 22.8 | 0.43 | 0.41 |
| **L2** | 20.3 | 0.42 | 0.49 |
| **L4** | 28.5 | 0.39 | 0.48 |
| **L5** | 24.1 | 0.42 | 0.46 |
| **L28** | 25.2 | 0.41 | 0.43 |
| **L43** | 18.6 | 0.41 | 0.48 |

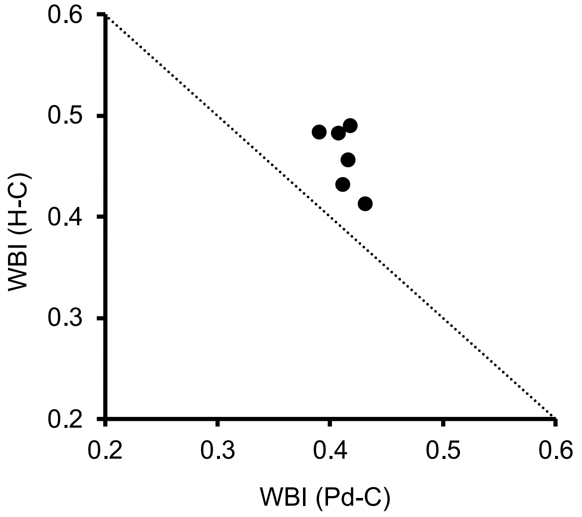


**Figure S4.** More O'Ferrall-Jencks analysis of calculated ligands.

## 7. NMR spectra

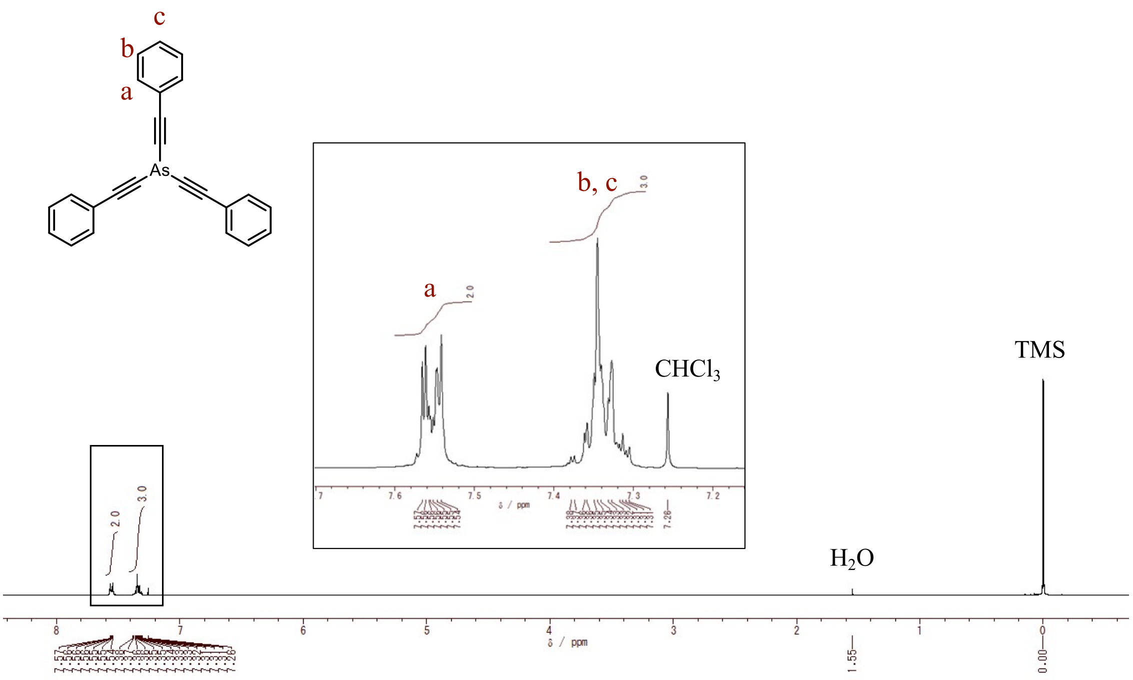


**Figure S5.** $^1$H-NMR spectrum (400 MHz) of **L3** in $CDCl_3$.

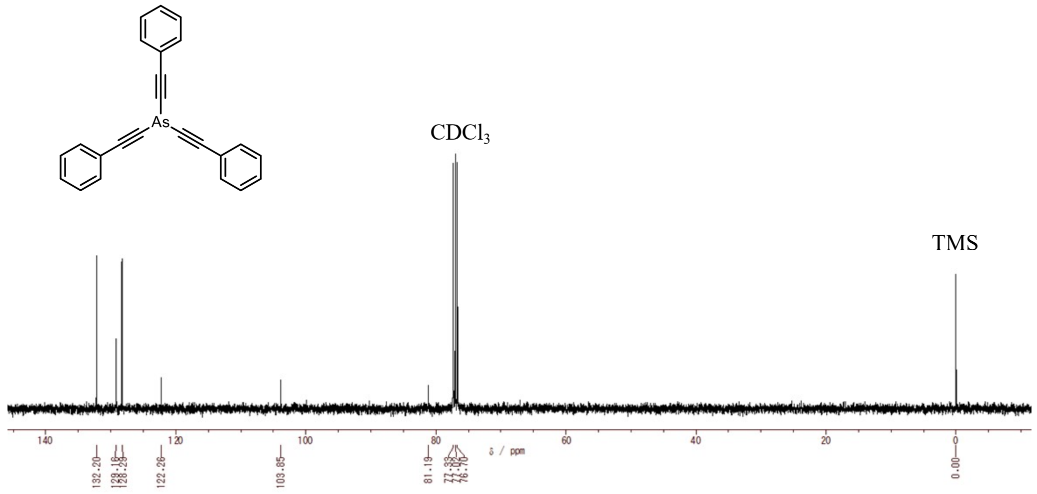


**Figure S6.** $^{13}C\{^{1}H\}$-NMR spectrum (100 MHz) of **L3** in $CDCl_3$.

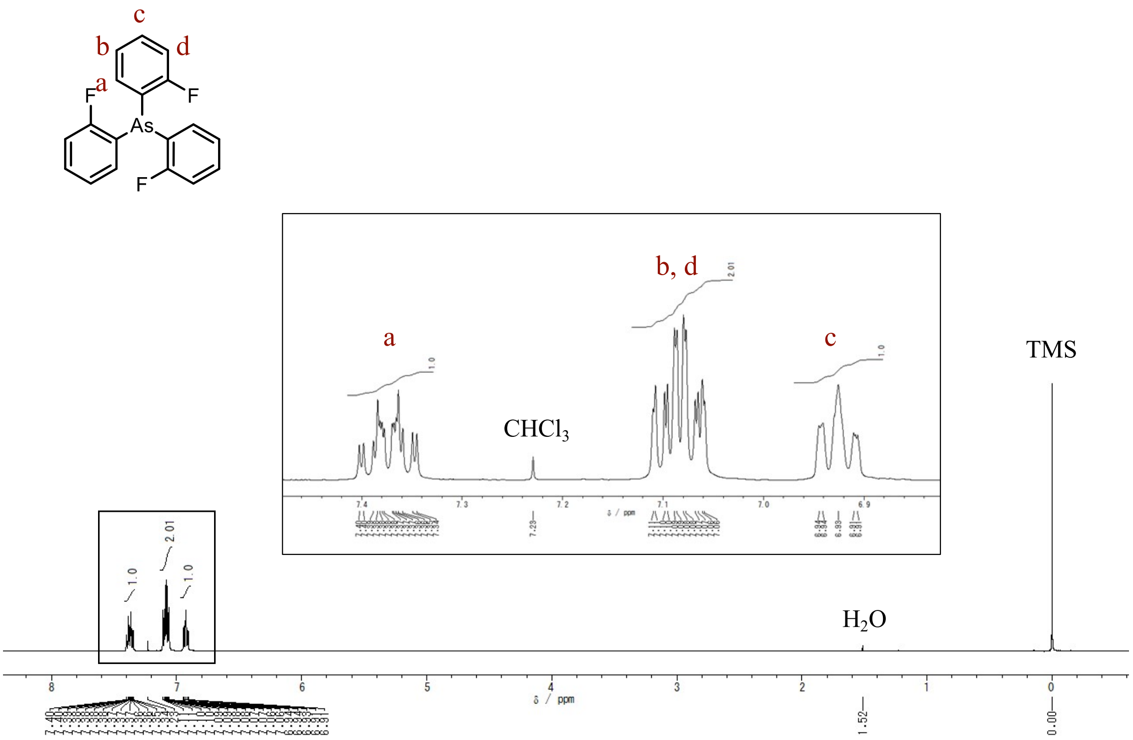


**Figure S7.** $^{1}H$-NMR spectrum (400 MHz) of **L5** in $CDCl_3$.

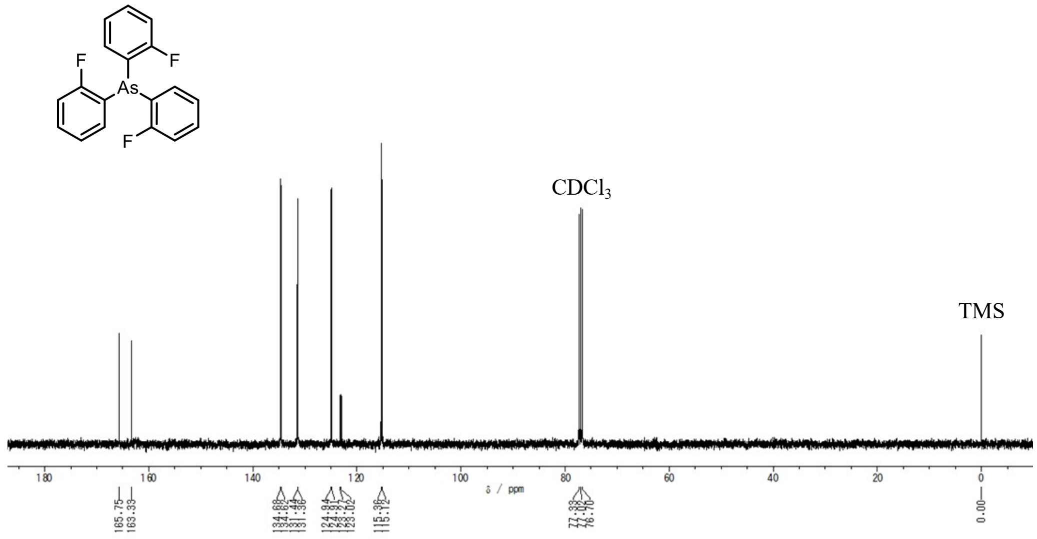


**Figure S8.** $^{13}C\{^{1}H\}$-NMR spectrum (100 MHz) of **L5** in $CDCl_3$.

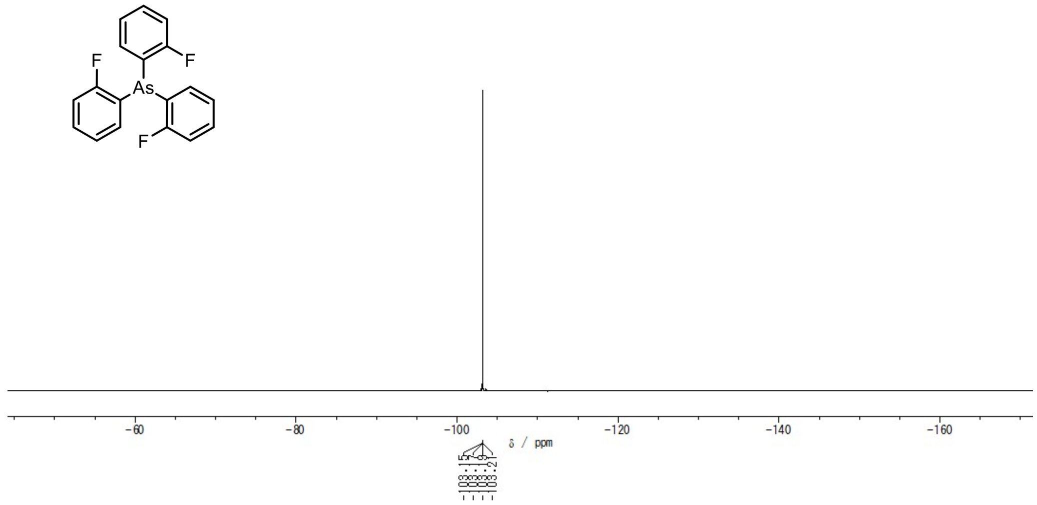


**Figure S9.** $^{19}F$-NMR spectrum (376 MHz) of **L5** in $CDCl_3$.

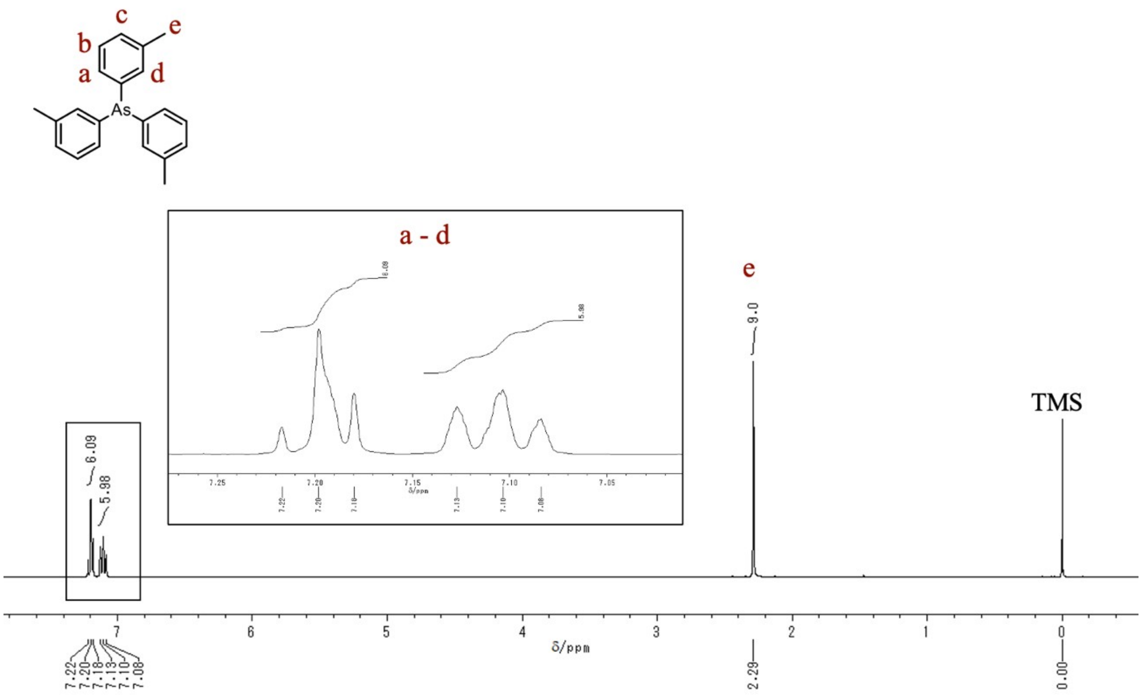


**Figure S10.** $^{1}$H-NMR spectrum (400 MHz) of **L8** in $CDCl_3$.

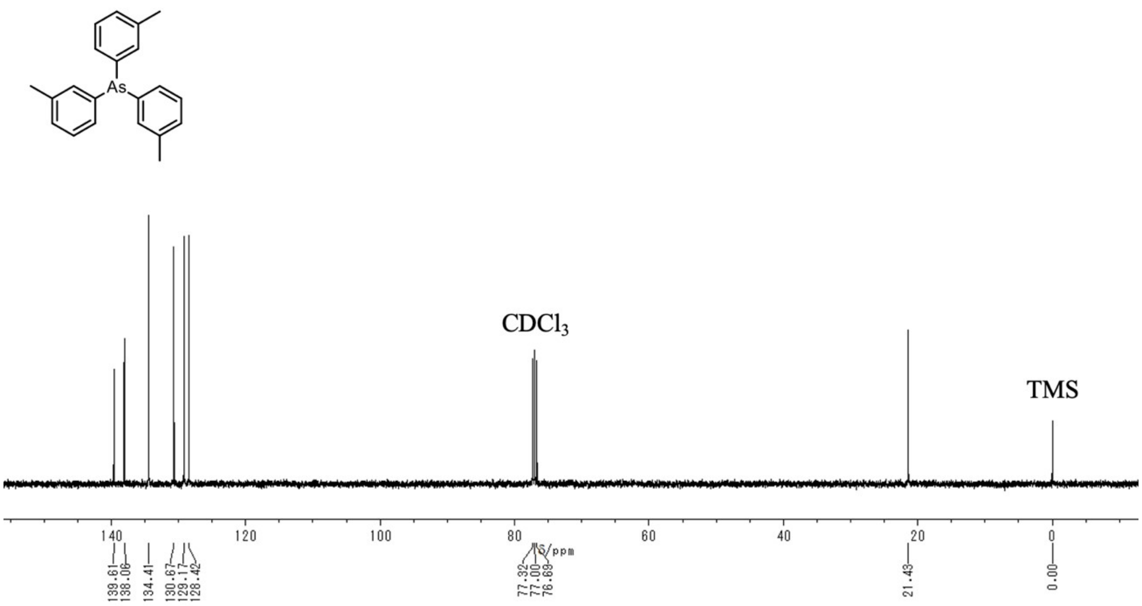


**Figure S11.** $^{13}$C{$^{1}$H}-NMR spectrum (100 MHz) of **L8** in $CDCl_3$.

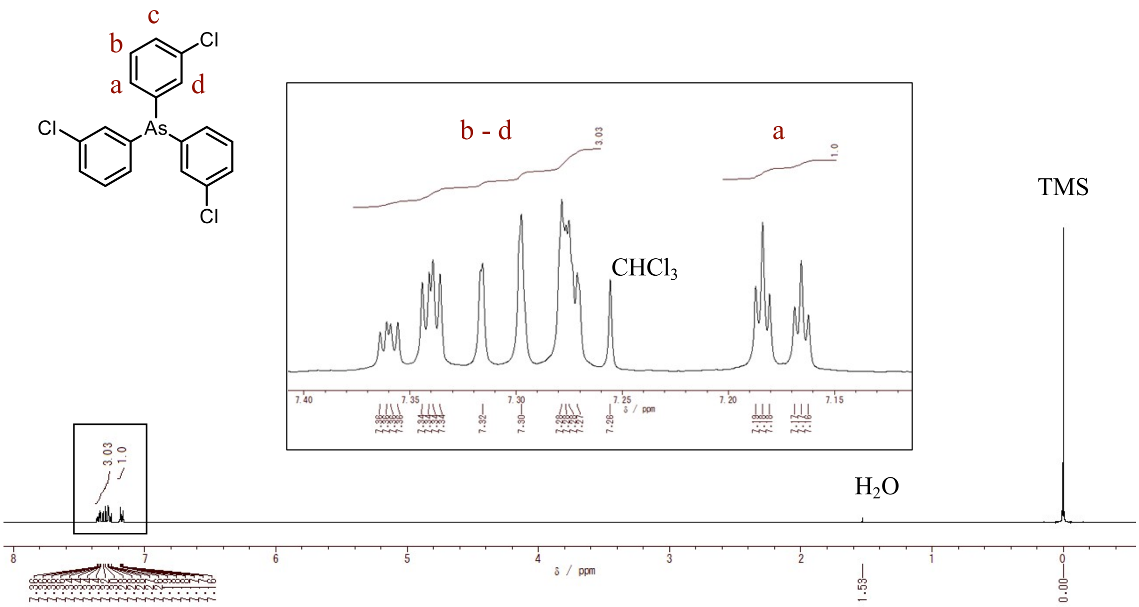


**Figure S12.** $^{1}$H-NMR spectrum (400 MHz) of **L9** in $CDCl_3$.

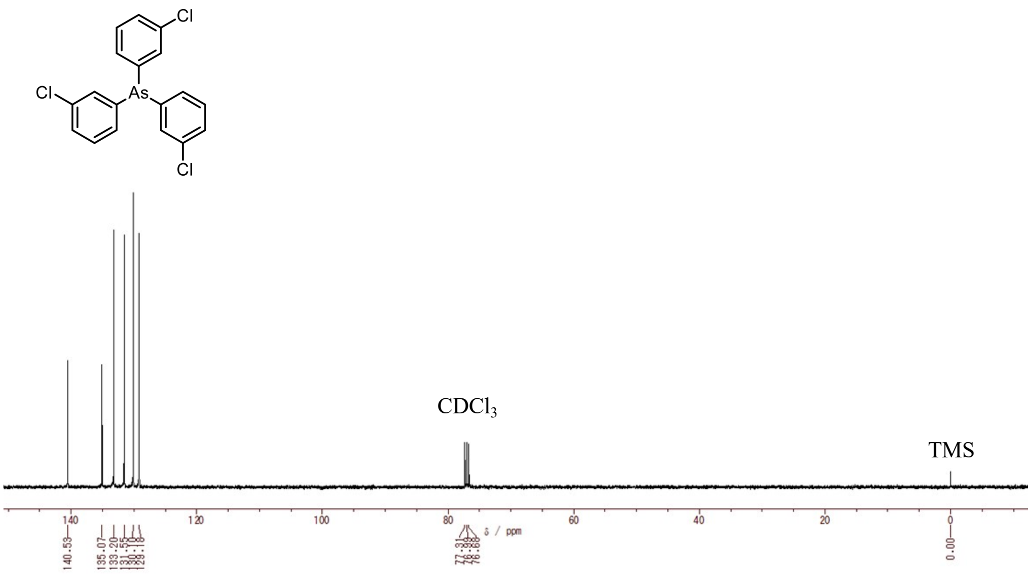


**Figure S13.** $^{13}C\{^{1}H\}$-NMR spectrum (100 MHz) of **L9** in $CDCl_3$.

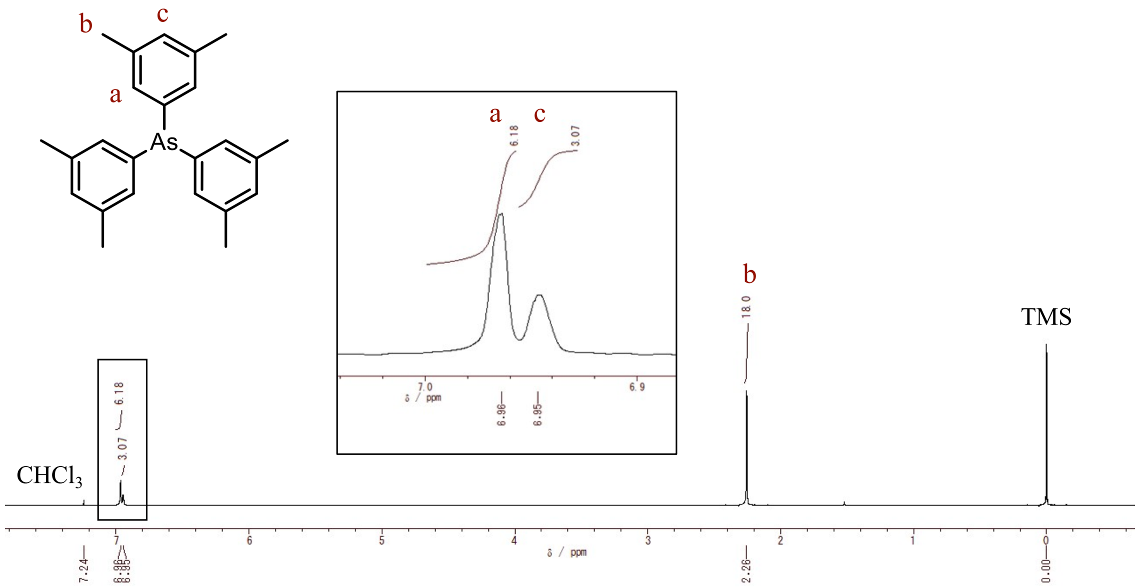


**Figure S14.** $^{1}$H-NMR spectrum (400 MHz) of **L10** in $CDCl_3$.

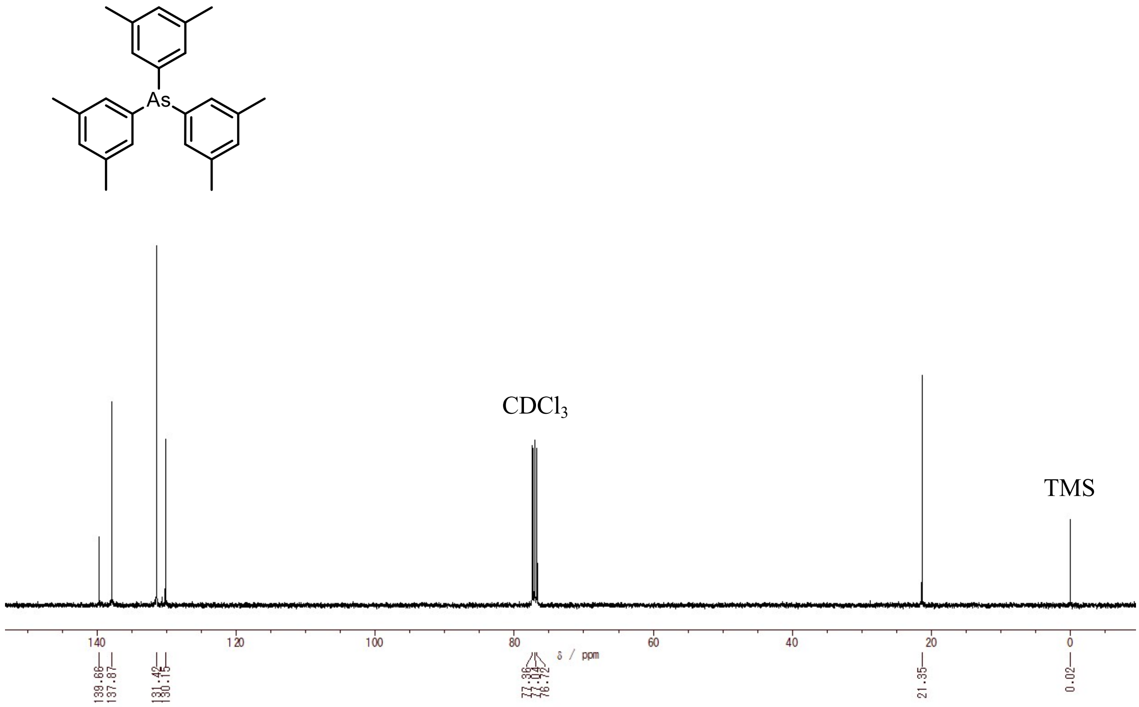


**Figure S15.** $^{13}C\{^{1}H\}$-NMR spectrum (100 MHz) of **L10** in $CDCl_3$.

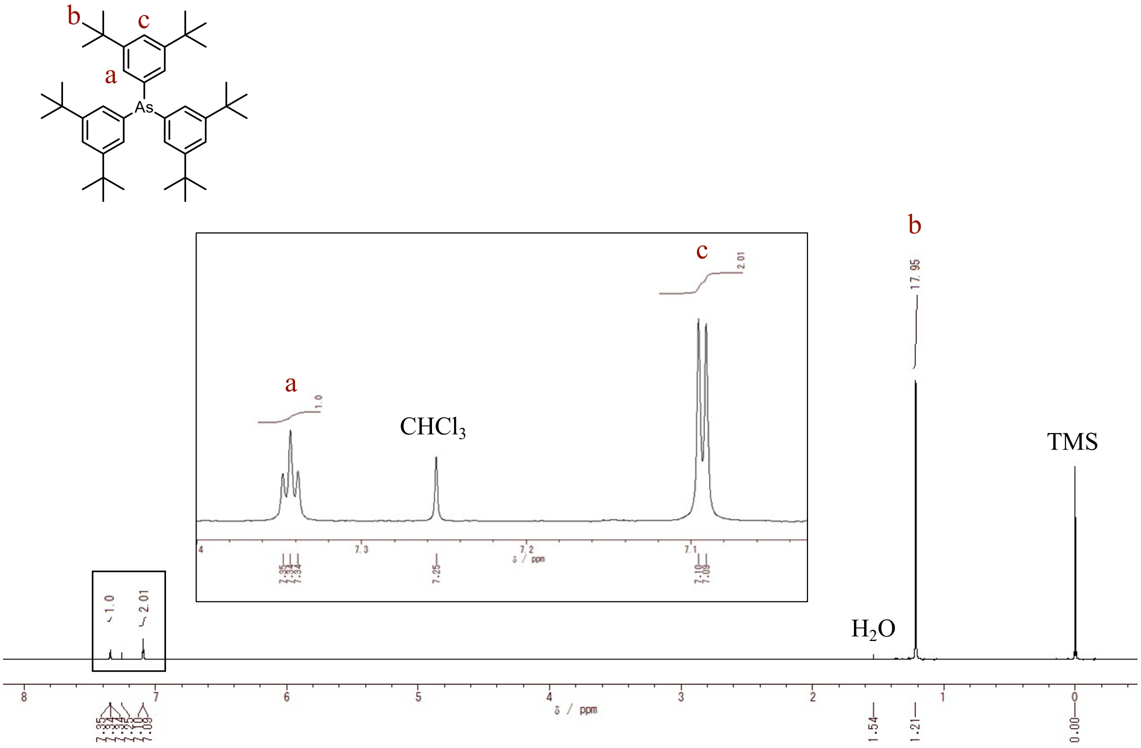


**Figure S16.** $^{1}$H-NMR spectrum (400 MHz) of **L11** in $CDCl_3$.

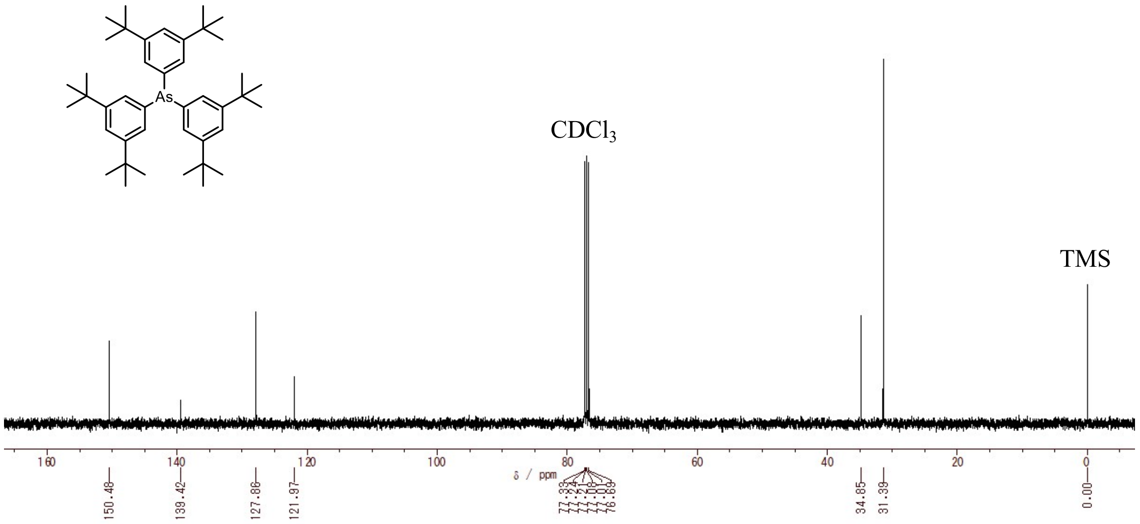


**Figure S17.** $^{13}$C{$^{1}$H}-NMR spectrum (100 MHz) of **L11** in $CDCl_3$.

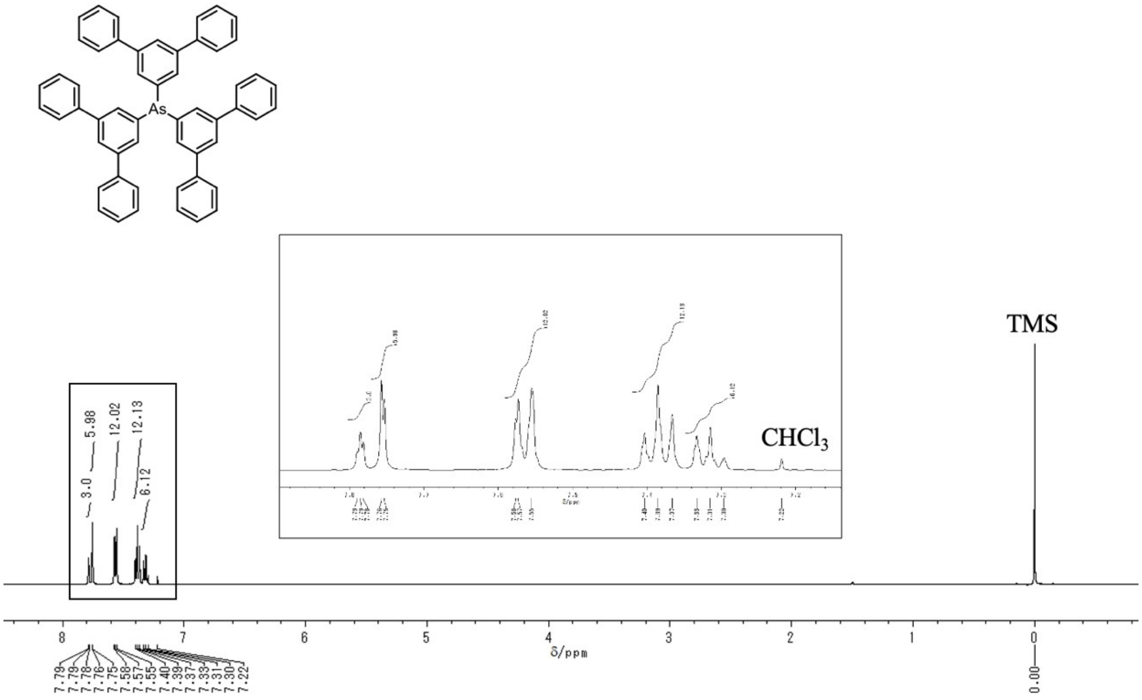


**Figure S18.** $^{1}$H-NMR spectrum (400 MHz) of **L12** in $CDCl_3$.

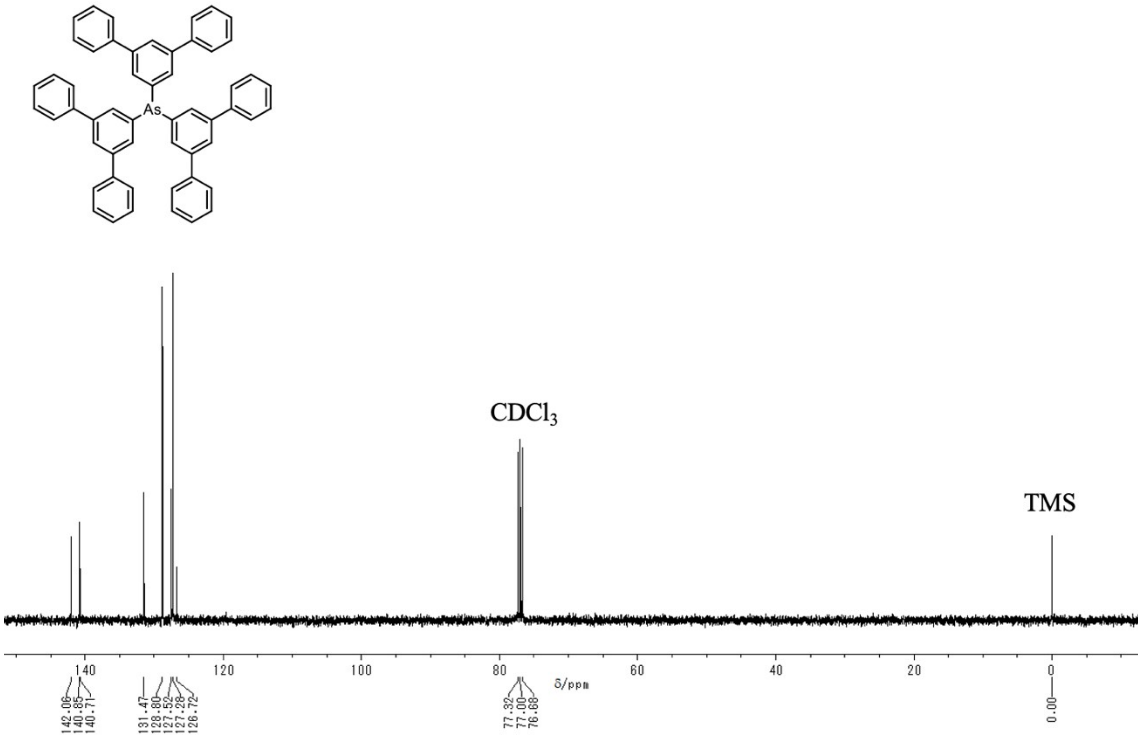


**Figure S19.** $^{13}$C{$^{1}$H}-NMR spectrum (100 MHz) of **L12** in $CDCl_3$.

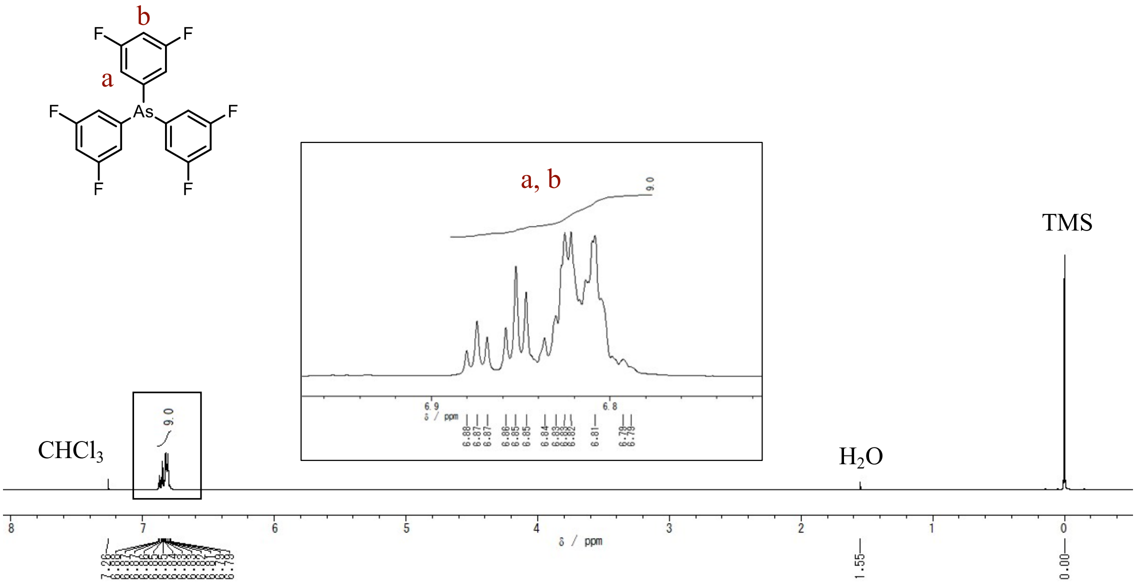


**Figure S20.** $^1$H-NMR spectrum (400 MHz) of **L13** in $CDCl_3$.

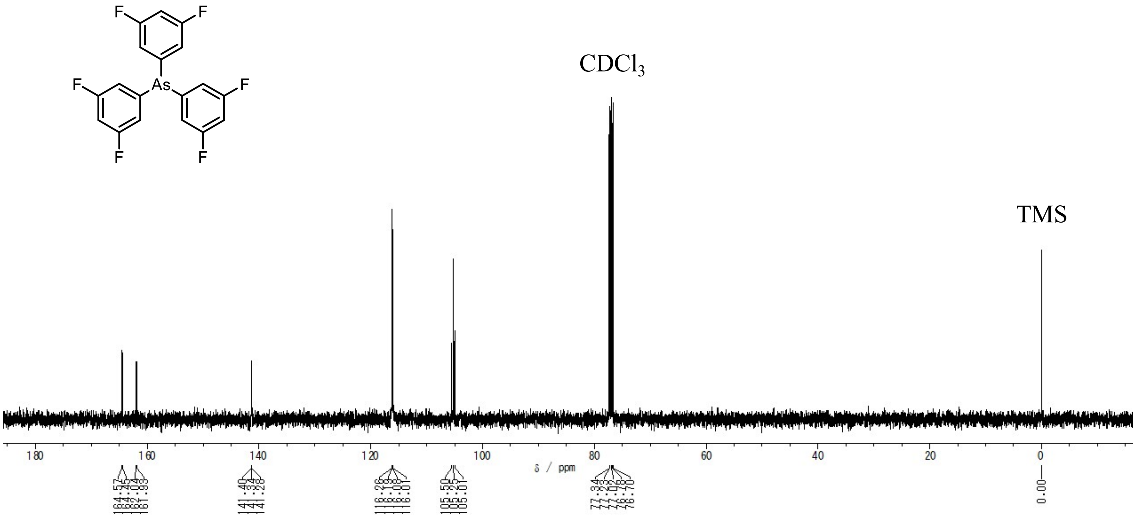


**Figure S21.** $^{13}C\{^1H\}$-NMR spectrum (100 MHz) of **L13** in $CDCl_3$.

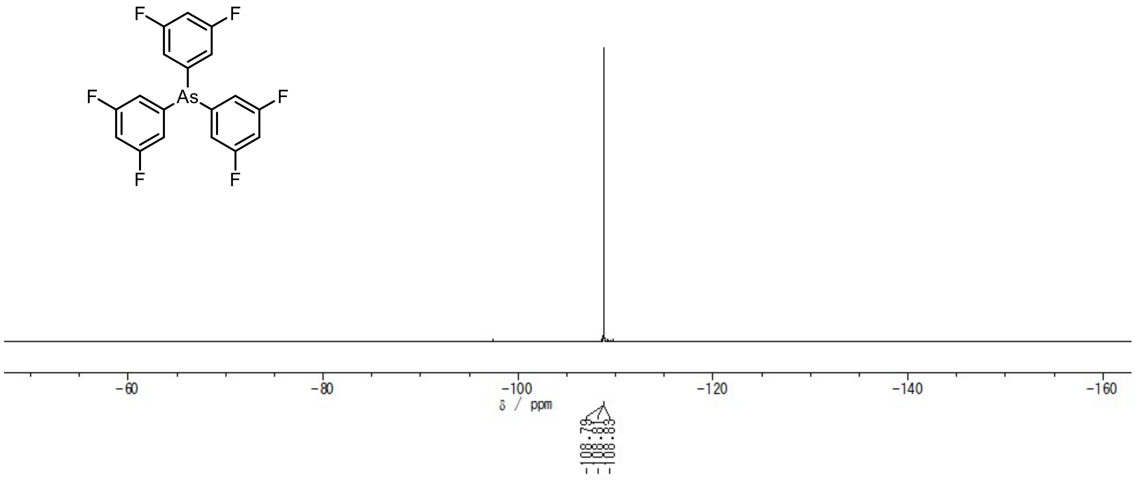

**Figure S22.** $^{19}$F-NMR spectrum (376 MHz) of **L13** in $CDCl_3$.

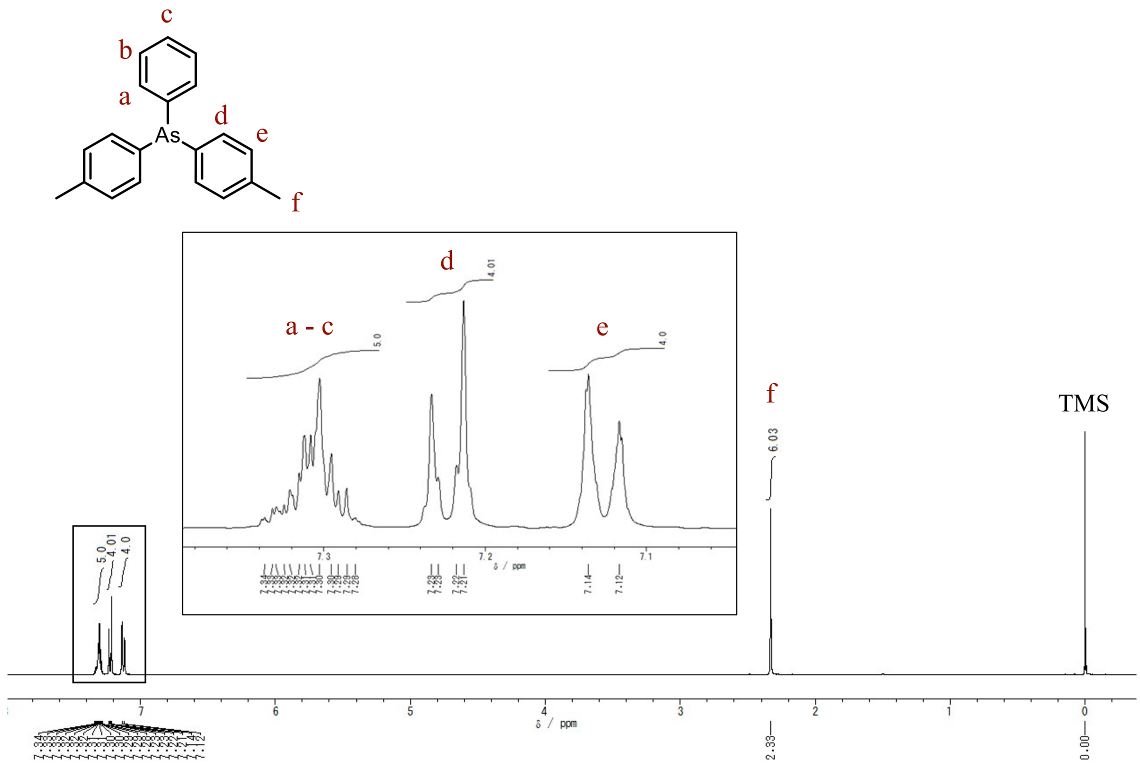

**Figure S23.** $^{1}$H-NMR spectrum (400 MHz) of **L37** in $CDCl_3$.

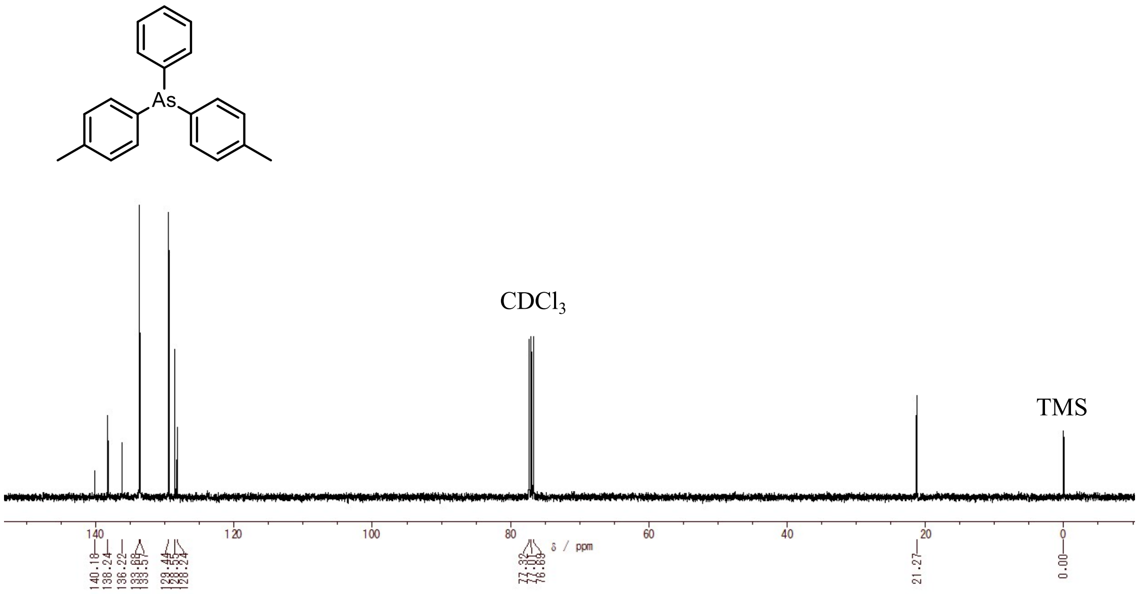


**Figure S24.** $^{13}C\{^1H\}$-NMR spectrum (100 MHz) of **L37** in $CDCl_3$.

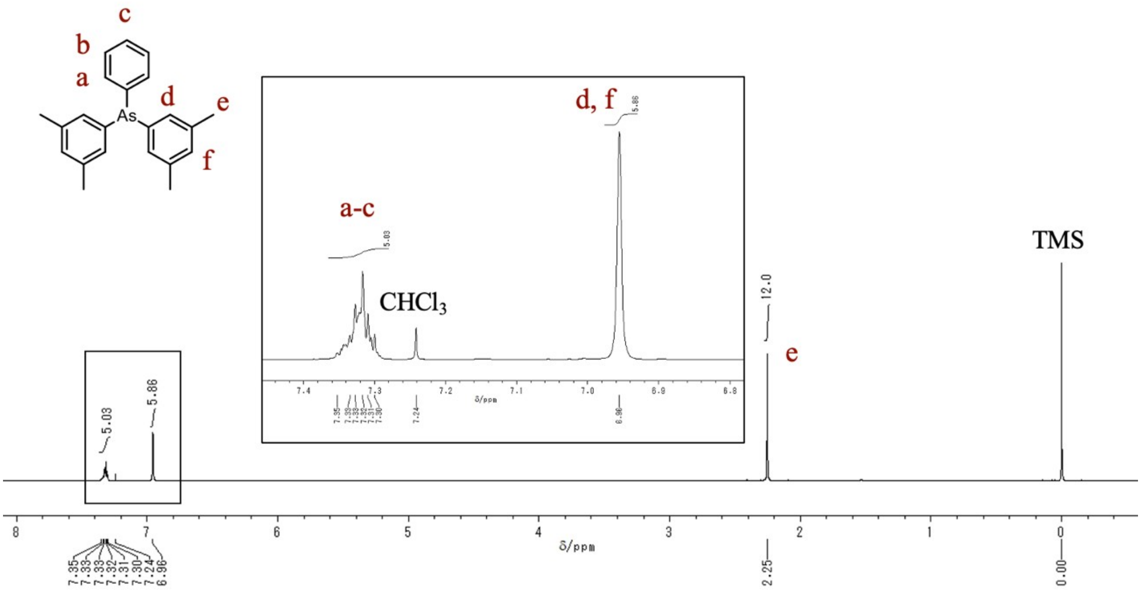


**Figure S25.** $^1H$-NMR spectrum (400 MHz) of **L38** in $CDCl_3$.

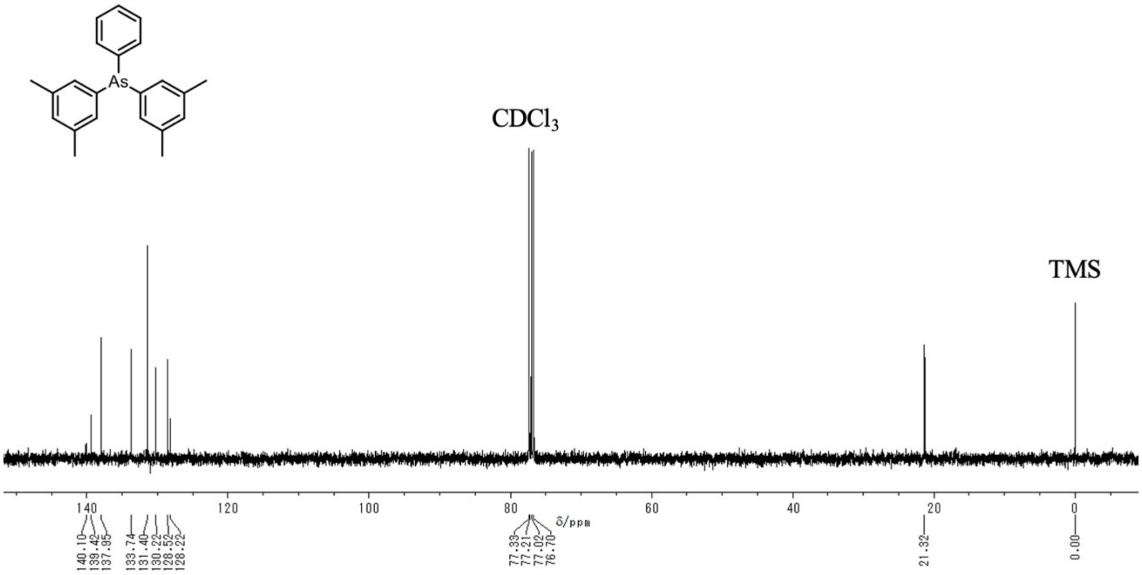


**Figure S26.** $^{13}C\{^{1}H\}$-NMR spectrum (100 MHz) of **L38** in $CDCl_3$.

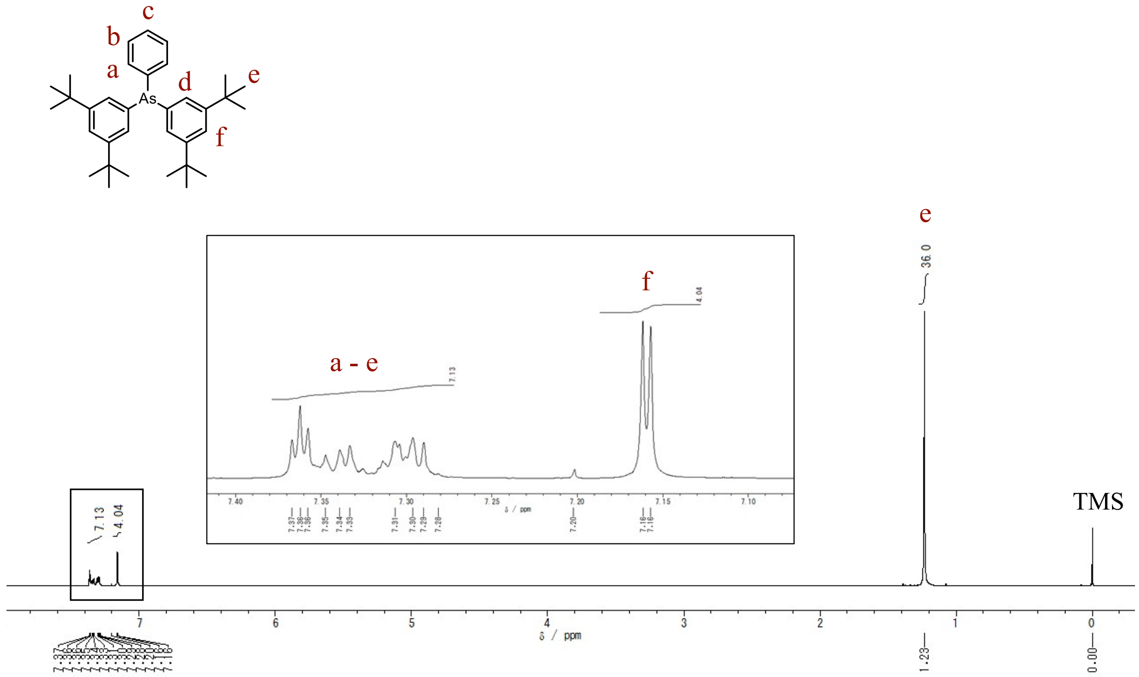


**Figure S27.** $^{1}H$-NMR spectrum (400 MHz) of **L39** in $CDCl_3$.

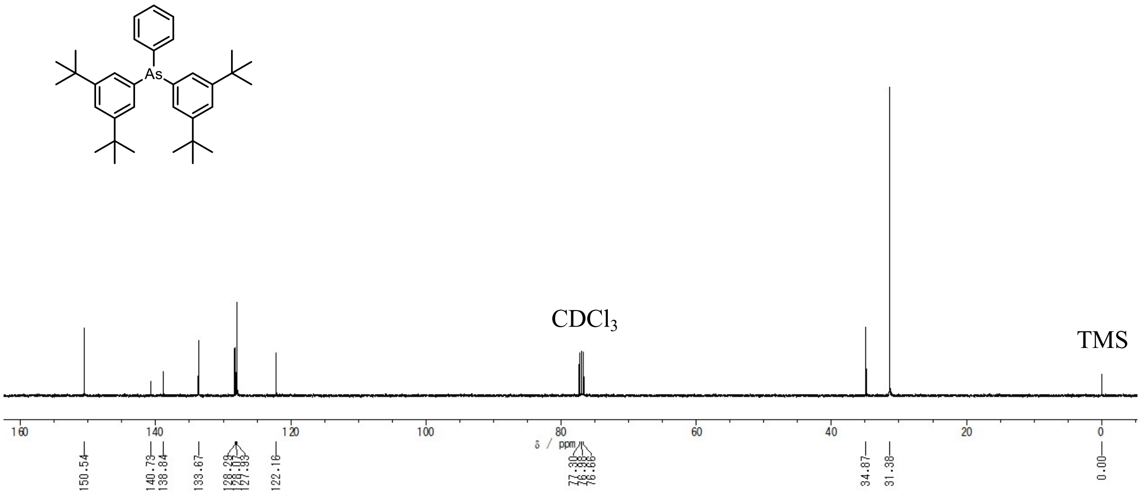


**Figure S28.** $^{13}C\{^1H\}$-NMR spectrum (100 MHz) of **L39** in $CDCl_3$.

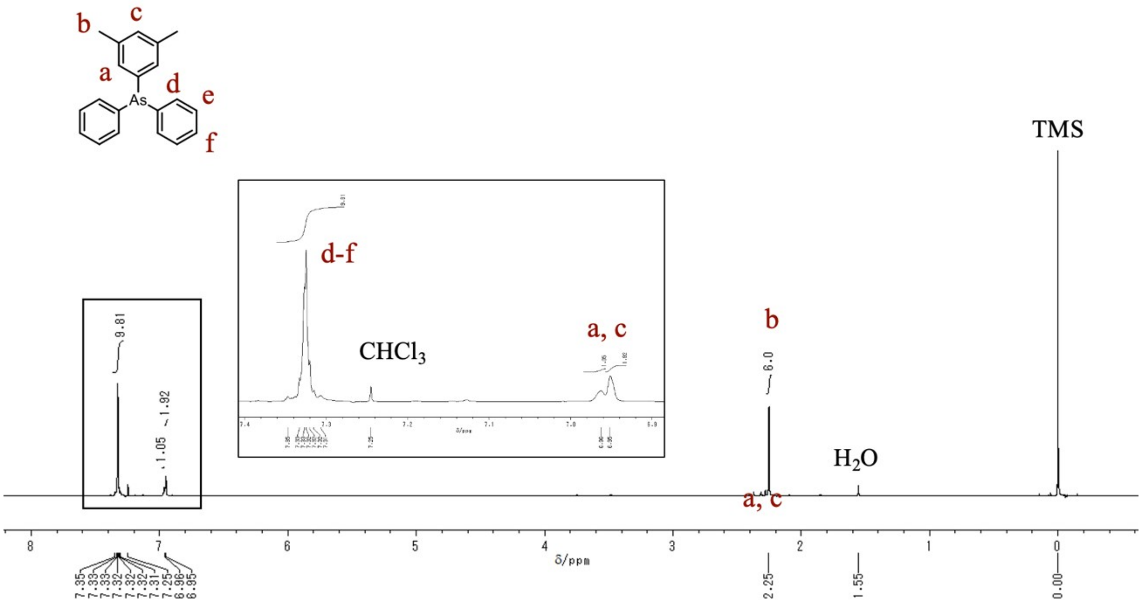


**Figure S29.** $^1H$-NMR spectrum (400 MHz) of **L40** in $CDCl_3$.

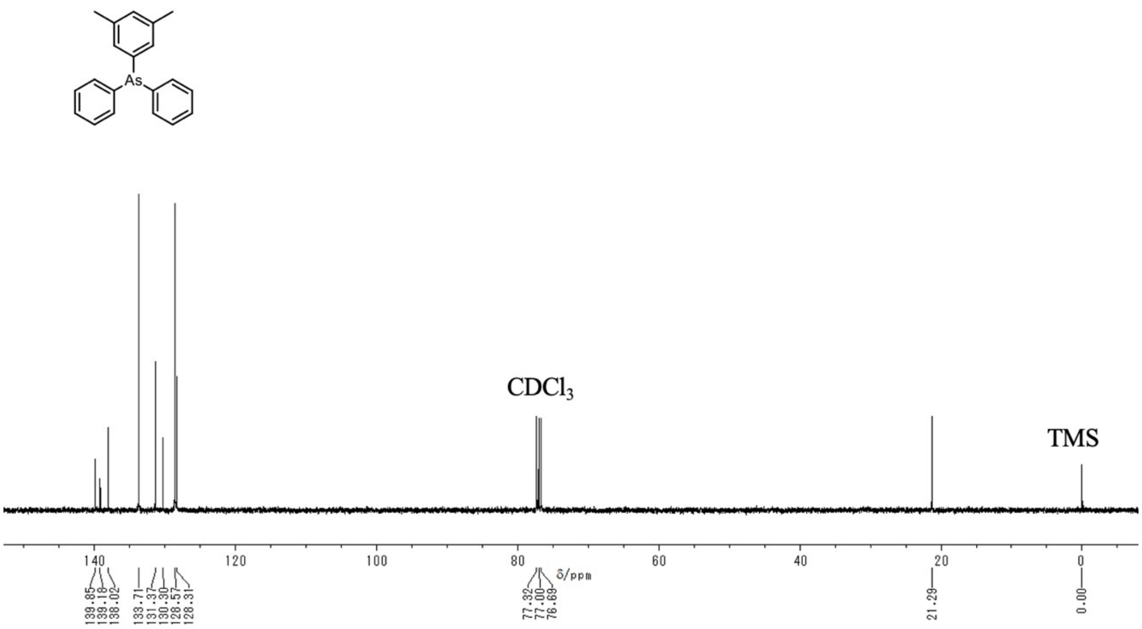


**Figure S30.** $^{13}C\{^{1}H\}$-NMR spectrum (100 MHz) of **L40** in $CDCl_3$.

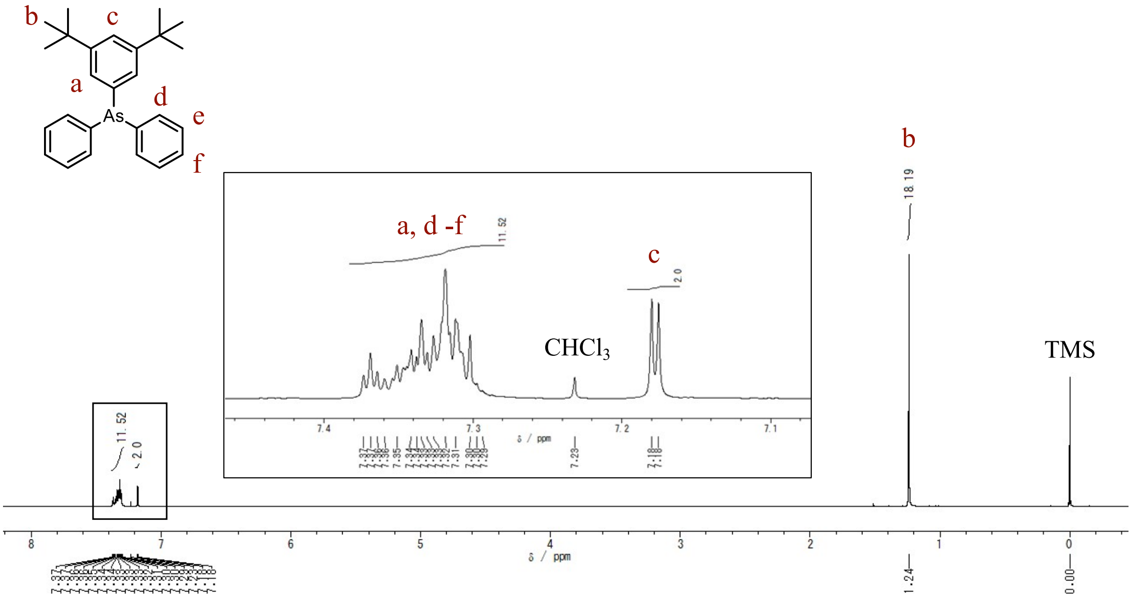


**Figure S31.** $^{1}H$-NMR spectrum (400 MHz) of **L41** in $CDCl_3$.

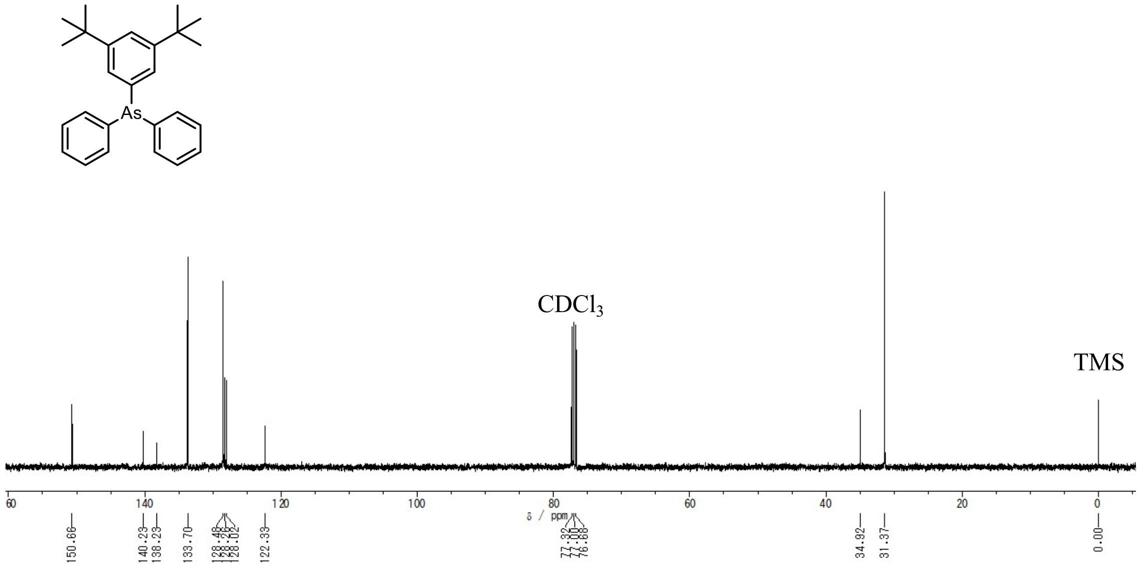


**Figure S32.** $^{13}C\{^{1}H\}$-NMR spectrum (100 MHz) of **L41** in $CDCl_3$.

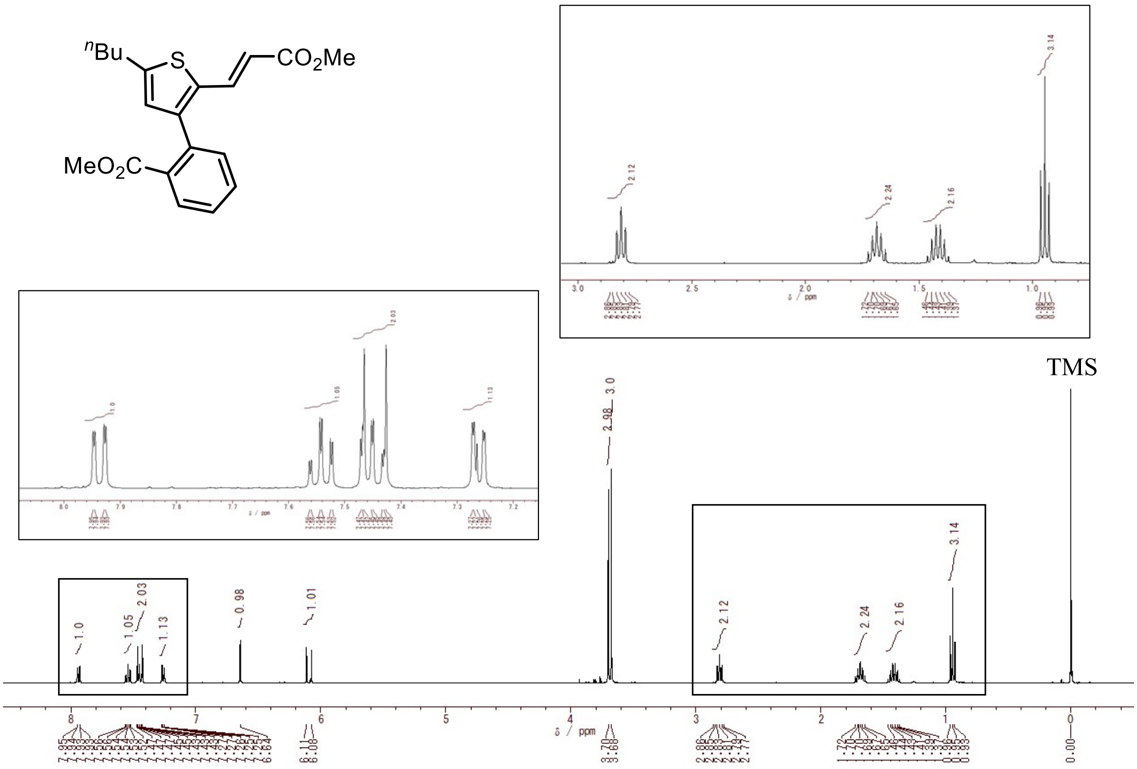


**Figure S33.** $^{1}H$-NMR spectrum (400 MHz) of **1** in $CDCl_3$.

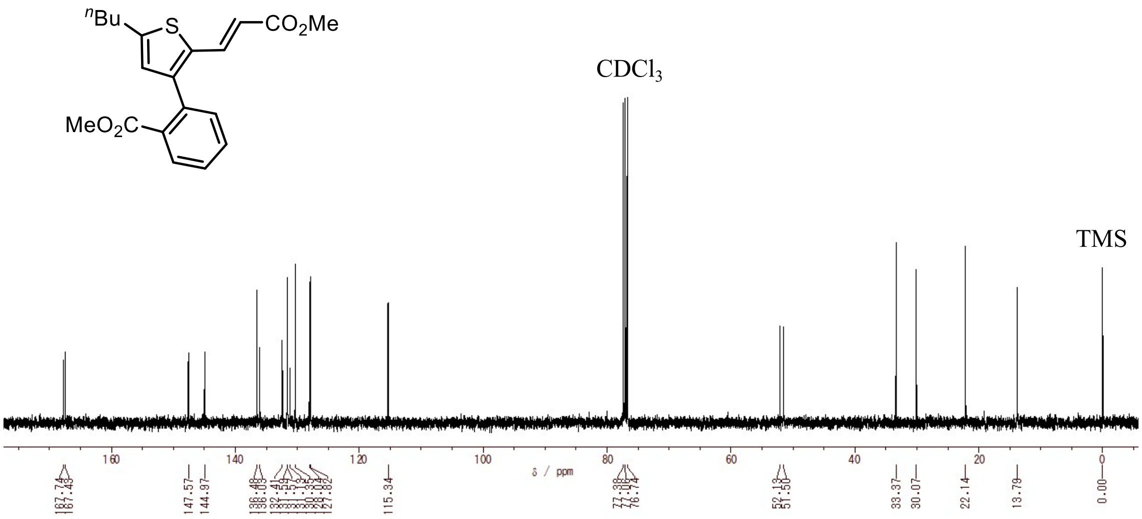


**Figure S34.** $^{13}C\{^1H\}$-NMR spectrum (100 MHz) of **1** in $CDCl_3$.

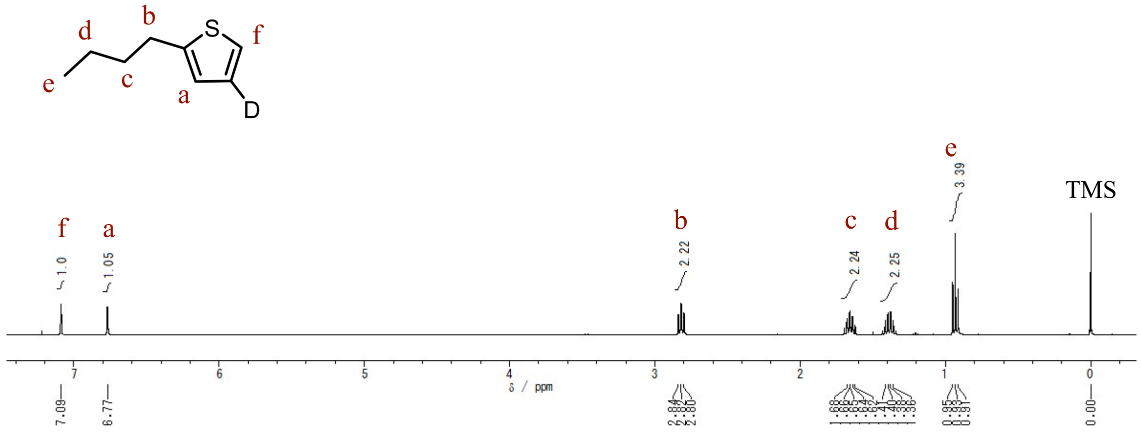


**Figure S35.** $^1H$-NMR spectrum (400 MHz) of *4-d-2-butylthiophene* in $CDCl_3$.

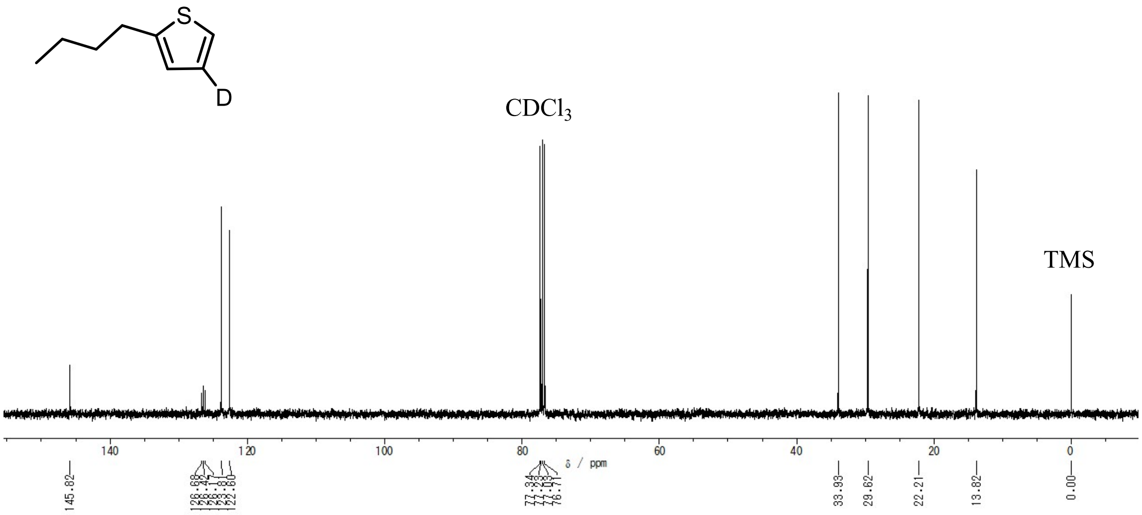


**Figure S36.** $^{13}C\{^1H\}$-NMR spectrum (100 MHz) of *4-d-2-butylthiophene* in $CDCl_3$.

## 8. References


1. M. S. Thomas, M. Landman, S. Lotz, Elizabeth M. van der Merwe, *Polyhedron* **2007**, *26,* 3471.
2. X. Song, H. Yu, Y. Zhang, Y. Miao, K. Ye, Y. Wang, *CrystEngComm* **2018**, *20,* 1669.
3. H. Zheng, D. G. Hall, *Tetrahedron Lett.* **2010**, *51*, 3561.
4. J. Wang, Z. Dong, C. Yang, G. Dong, *Nat. Chem*. **2019**, *11,* 1106.
5. J. W. B. Reesor, G. F. Wright, *J. Org. Chem*. **1957**, *22*, 382.
6. S. Tanaka, M. Konishi, H. Imoto, Y. Nakamura, M. Ishida, H. Furuta, K. Naka, *Inorg. Chem.* **2020**, *59,* 9587.
7. J. W. Dube, Y. Zheng, W. Thiel, M. Alcarazo, *J. Am. Chem. Soc.* **2016**, *138,* 6869.
8. A. Chishiro, M. Konishi, R. Inaba, T. Yumura, H. Imoto, K. Naka, *Dalton. Trans.* **2022**, *51,* 95.
9. J. Yukiyasu, R. Inaba, T. Yumura, H. Imoto, K. Naka, *Org. Chem. Front.* **2022**, *9,* 6786.
10. A. Sumida, H. Imoto, K. Naka, *Inorg. Chem.* **2022**, *61,* 17419.
11. T. Kato, S. Tanaka, K. Naka, *Chem. Lett.* **2015**, *44,* 1476.
12. T. Fujii, A. Urushizaki, H. Imoto, K. Naka, *Asian. J. Org. Chem.* **2023**, *12,* e202300067.
13. J. Yukiyasu, A. Sumida, H. Shimoji, A. Urushizaki, T. Yanagihara, H. Ogoshi, Y. Nakamura, S. Ito, K. Tanaka, H. Imoto, K. Naka, *Eur. J. Inorg. Chem.* **2024**, *27,* e202400036.
14. A. G. Dalling, T. Yamauchi, N. G. McCreanor, L. Cox, J. F. Bower, *Angew. Chem. Int.*

*Ed.* **2019**, *58,* 221.

15. G. J. Quinteros, P. M. Uberman, S. E. Martin, *Eur. J. Org. Chem.* **2015**, *12,* 2698.
16. R. Li, Y. Zhou, X. Xu, G. Dong, *J. Am. Chem. Soc.* **2019**, *141*, 18958.
17. Y. L. Luo, H. Z. Dua, B.T. Guan, *Org. Chem. Front.* **2021**, *8,* 4171.
18. J. Palaty, F. S.Abbott, *J. Med. Chem.* **1995**, *38,* 3398.
